# Live‐Cell Localization Microscopy with a Fluorogenic and Self‐Blinking Tetrazine Probe

**DOI:** 10.1002/anie.201906806

**Published:** 2019-11-18

**Authors:** Philipp Werther, Klaus Yserentant, Felix Braun, Nicolai Kaltwasser, Christoph Popp, Mathis Baalmann, Dirk‐Peter Herten, Richard Wombacher

**Affiliations:** ^1^ Institut für Pharmazie und Molekulare Biotechnologie Ruprecht-Karls-Universität Heidelberg Im Neuenheimer Feld 364 69120 Heidelberg Germany; ^2^ Physikalisch-Chemisches Institut Ruprecht-Karls-Universität Heidelberg Im Neuenheimer Feld 229 69120 Heidelberg Germany; ^3^ CellNetworks, Single-Molecule Spectroscopy Ruprecht-Karls-Universität Heidelberg Im Neuenheimer Feld 267 69120 Heidelberg Germany; ^4^ Fakultät für Biowissenschaften Ruprecht-Karls-Universität Heidelberg Im Neuenheimer Feld 234 69120 Heidelberg Germany; ^5^ Institute of Cardiovascular Sciences & School of Chemistry College of Medical and Dental Sciences Medical School University of Birmingham Edgbaston Birmingham B15 2TT UK; ^6^ Centre of Membrane Proteins and Receptors (COMPARE) Universities of Birmingham and Nottingham Midlands UK

**Keywords:** bioorthogonal chemistry, click chemistry, fluorescent probes, super-resolution imaging, tetrazines

## Abstract

Recent developments in fluorescence microscopy call for novel small‐molecule‐based labels with multiple functionalities to satisfy different experimental requirements. A current limitation in the advancement of live‐cell single‐molecule localization microscopy is the high excitation power required to induce blinking. This is in marked contrast to the minimal phototoxicity required in live‐cell experiments. At the same time, quality of super‐resolution imaging depends on high label specificity, making removal of excess dye essential. Approaching both hurdles, we present the design and synthesis of a small‐molecule label comprising both fluorogenic and self‐blinking features. Bioorthogonal click chemistry ensures fast and highly selective attachment onto a variety of biomolecular targets. Along with spectroscopic characterization, we demonstrate that the probe improves quality and conditions for regular and single‐molecule localization microscopy on live‐cell samples.

## Graphical abstract

**Blink and you'll miss it**: A rationally designed small‐molecule label featuring fluorogenic and self‐blinking features has been designed and synthesized for bioorthogonal chemistry and super‐resolution microscopy in living cells.

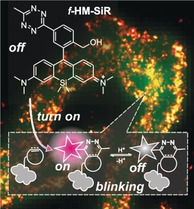

## Introduction

Small‐molecule fluorophores are essential tools for biological imaging, which is a key method in modern life sciences. With the rapid development of novel fluorescence‐based imaging techniques, the design and chemical synthesis of fluorophores with improved photophysical properties has experienced an enormous revival.^1^ In particular, super‐resolution imaging techniques, such as single‐molecule localization microscopy (SMLM), like direct stochastic optical reconstruction microscopy (dSTORM) or photoactivated localization microscopy (PALM), are heavily dependent upon individual properties of the applied organic fluorophores.^2^ Active control of emissive and dark states required for the localization of individual emitters in SMLM typically requires high intensity illumination, addition of redox reagents or UV exposure, rendering SMLM in a live specimen challenging.^3^ Spontaneously blinking fluorescent probes^4^ based on the silicon rhodamine (SiR) scaffold^5^ have recently been developed to enable live‐cell SMLM under physiological conditions. In this approach, a hydroxymethyl nucleophile causes the reversible intramolecular formation of a non‐chromophoric spiroether. This chemical equilibrium of hydroxymethyl SiR (HM‐SiR) leads to stochastically occurring transitions between the excitable quinoid and the non‐excitable spiroether isomer. Hence, strenuous manipulation of the fluorophore excited state to populate dark states is not required. This enables SMLM recordings without additional stimulation of on–off transitions.

The quality of image reconstruction in SMLM is governed by blinking kinetics, labeling density, and signal specificity.^6^ In STORM and PALM the first is controlled by adapting excitation power and redox buffer properties. In contrast, blinking kinetics of spontaneously blinking fluorophores is an intrinsic property that can be influenced by a change in the local microenvironment; Localizing HM‐SiR to lipid membranes, for instance, has resulted in improved performance in SMLM.^7^ Regarding labeling density and signal specificity, fast, quantitative, and highly selective labeling reactions with a minimum of unspecific binding are crucial. Among bioconjugation methods, bioorthogonal reactions such as the inverse electron demand Diels Alder reaction (DA_inv_) between 1,2,4,5‐tetrazines and ring‐strained alkenes and alkynes have lately received significant attention. DA_inv_ is particularly popular due to its fast kinetics, chemoselectivity, and biocompatibility, making it well‐suited for live‐cell applications.^8^ Therefore, tetrazine‐modified small‐molecule fluorophores have a broad applicability as they can be installed at virtually any dienophile‐tagged biomolecule. They allow specific labeling in combination with protein tags,^9^ peptide tags,^10^ and unnatural amino acids,^11^ pushing the label size to a minimum. Moreover, DA_inv_ enables labeling various biomolecules other than proteins like nucleotides,^12^ sugars,^13^ and lipids^14^ as well as to exploit small‐molecule‐mediated targeting.^15^ Additionally, tetrazine‐based labeling can be used to reduce unspecific signal; Carefully designed tetrazine probes have been shown to undergo an increase in fluorescence when the tetrazine is consumed in DA_inv_, that is, when the dye label is covalently linked to its target structure.15a, 16 This is a valuable additional feature for live‐cell fluorescence microscopy because it obviates the need for extensive excess dye wash‐out and dramatically reduces background signal.^17^ This is particularly advantageous for SMLM as the localization precision is affected by background signal.^18^ Overall, we reason that a broadly applicable probe for SMLM would comprise all of the above‐mentioned features. It should be as small as possible, self‐blinking, fluorogenic, readily suited for bioconjugation, and equipped with generally favorable photophysical properties, such as high brightness and photostability.

Here, we report the first merger of all these properties in the fluorogenic, far red‐emitting, self‐blinking silicon rhodamine ***f*** **‐HM‐SiR**. This tetrazine‐derivatized HM‐SiR is initially strongly quenched and shows fluorescence enhancement upon bioconjugation in DA_inv_. We report the synthesis and photophysical properties of the novel fluorogenic dye and demonstrate its application in live‐cell super‐resolution microscopy. The tetrazine functionalization was utilized to attach ***f*** **‐HM‐SiR** to intracellular targets by fast and selective click chemistry in living cells and to visualize intracellular dynamics by SMLM. Importantly, the fluorogenicity of ***f*** **‐HM‐SiR** allows for minimal‐ or no‐wash procedures in live‐cell imaging while its self‐blinking feature minimizes phototoxicity in SMLM experiments.

## Results and Discussion

**Figure 1:**
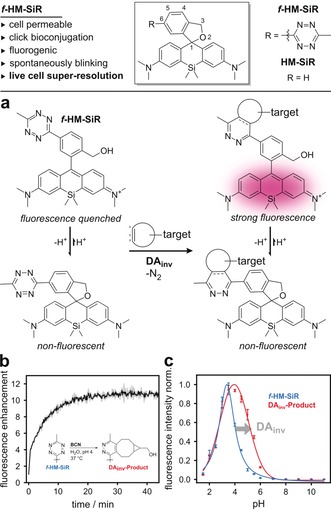
***f*** **‐HM‐SiR**, a fluorogenic and spontaneously blinking fluorophore for bioorthogonal DA_inv_. a) Spiro‐cyclization equilibrium of spontaneously blinking and fluorogenic ***f*** **‐HM‐SiR** and its Diels–Alder cycloadduct (**DA_inv_‐Product**). b) Fluorescence enhancement of ***f*** **‐HM‐SiR** upon conversion in DA_inv_ at pH 4 in aqueous buffer. c) pH‐dependent fluorescence of ***f*** **‐HM‐SiR** (blue) and **DA_inv_‐Product** (red).

First, we examined synthetic strategies to access fluorophore probes that combine both tetrazine and the desired structural hydroxymethyl motif in the SiR scaffold. Synthesis with tetrazines requires mild reaction conditions as they are highly base sensitive and react with strong nucleophiles. Therefore, a mild Lewis‐acid‐mediated Friedel–Crafts reaction16e was selected as the key step for the assembly of the HM‐SiR derivative. We set out to test the feasibility of this strategy for the synthesis of the simple unfunctionalized HM‐SiR without tetrazine (Figure 1) and found that this four step route using methoxymethyl (MOM) protection group chemistry enabled the synthesis of **HM‐SiR** at 22 % overall yield (Supporting Information, Scheme S2). This approach complements previous strategies relying on the addition of aryl lithium reagents to Si‐anthrones4a and could offer a short and mild synthesis route for similar fluorophores in the future. Our attention then turned to the synthesis of the tetrazine‐modified HM‐SiR derivative, in the following termed fluorogenic HM‐SiR (***f*** **‐HM‐SiR**). For this purpose, we synthesized the appropriate tetrazinyl benzaldehyde (**5**, Supporting Information, Scheme S1) as electrophile for the Friedel–Crafts reaction. Conversion with the diarylsilane (**6**, Supporting Information, Scheme S1) gave access to the desired ***f*** **‐HM‐SiR**, which carries both a 3‐hydroxyl and a 6‐tetrazine substituent (Figure 1). Both substituents are crucial for the intramolecular modulation of the probe's spectral properties: While fluorescence of ***f*** **‐HM‐SiR** is strongly quenched by the tetrazine moiety (Figure 1 a), it is restored by conversion with dienophile‐equipped biomolecular targets in DA_inv_ (Figure 1 a,b). The resulting Diels–Alder product of ***f*** **‐HM‐SiR** still exhibits the pH‐dependent equilibrium between absorbing (open) and non‐absorbing form (closed spiroether) and thereby spontaneously switches between fluorescent and non‐fluorescent states. Consequently, external control of the emitter density, which is otherwise essential in SMLM, becomes obsolete.

In order to evaluate the fluorogenicity of ***f*** **‐HM‐SiR** in DA_inv_, it was treated with bicyclo[6.1.0]non‐4‐yne (BCN) dienophile, which resulted in a 10‐fold fluorescence enhancement upon reaction (Figure 1 b). Furthermore, we determined a substantial increase in brightness of the respective isolated cycloadduct **DA_inv_‐Product** (Supporting Information, Table S1). To assess the self‐blinking properties of ***f*** **‐HM‐SiR**, we studied the reversible spirocyclization reaction. Both, the quinoid and the spiroether isomer of ***f*** **‐HM‐SiR**, could be observed in ^1^H NMR (Supporting Information, Figure S3). The pH‐dependent equilibrium between the two isomers was investigated by absorbance (Supporting Information, Figure S2) and fluorescence (Figure 1 c) spectroscopy. Here, we determined an equilibrium constant of p*K*_cycl_=4.0(±0.1), indicating that the non‐fluorescent spiroether form of ***f*** **‐HM‐SiR** significantly prevails at physiological conditions (99.9 %).4a The isolated **DA_inv_‐Product** showed a significant shift to p*K*_cycl_=5.2(±0.1), indicating that the proportion of the fluorescent quinoid form of the target‐bound ***f*** **‐HM‐SiR** is increased but remains below 1 % at physiological pH (99.4 % spiroether). The higher abundance of the quinoid isomer of target‐bound ***f*** **‐HM‐SiR** infers that the emissive state will be more populated compared to unreacted dye leading to an overall reduction of unspecific signal. Consequently, two mechanisms of fluorogenicity could be observed: one corresponds to the loss of the quenching tetrazine, the other to the changed tendency of the product to form the spiroether. Following up on those results, we set out to evaluate the general suitability of ***f*** **‐HM‐SiR** for bioconjugation. A purified HaloTag‐EGFP was modified with HaloTag ligand‐BCN (HTL‐BCN) and subsequently reacted with ***f*** **‐HM‐SiR**. In‐gel fluorescence showed specific labeling, while the pH‐dependent emission of the EGFP‐bound dye was retained (Supporting Information, Figure S7).

**Figure 2:**
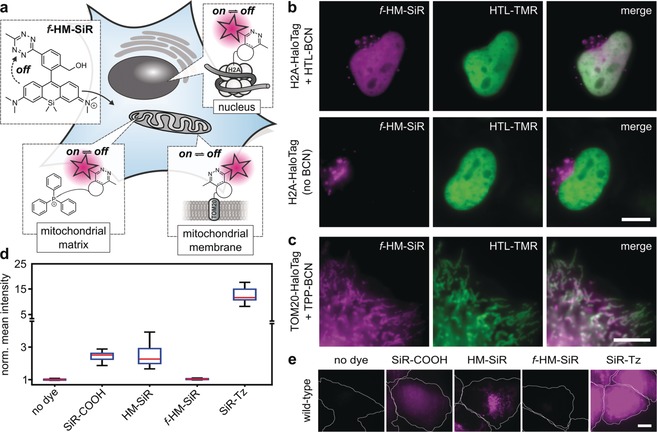
Cell‐permeable ***f*** **‐HM‐SiR** can be specifically conjugated in live cells by DA_inv_. a) Prior to reaction with dienophile, ***f*** **‐HM‐SiR** resides in quenched form. Target‐bound ***f*** **‐HM‐SiR** spontaneously switches between fluorescent on‐ and off‐form. b) HeLa cells transiently expressing H2A‐HaloTag were incubated with (upper row) or without (lower row) HTL‐BCN (10 μm) for 30 min, washed, and labeled with ***f*** **‐HM‐SiR** (2 μm, magenta) prior to imaging. Reference images (green) were generated by labeling with HTL‐TMR after incubation with HTL‐BCN. c) HeLa cells transiently expressing TOM20‐HaloTag were incubated with TPP‐BCN (10 μm) and HTL‐TMR (green), washed, and labeled with ***f*** **‐HM‐SiR** (2 μm, magenta) prior to imaging. d) Background intensity of HeLa cells incubated with various SiR derivatives (2 μm), *n*=20. Medium was replaced once after incubation with dyes. Note the broken *y*‐axis and change of scale. e) Representative images for diagram in (d). Settings for image acquisition, processing, and display were identical for all shown images. Outlines of cells indicated by white line. Scale bars: b,c,e) 10 μm.

Based on these promising photophysical characteristics, we moved on to apply the probe in live‐cell wide‐field fluorescence imaging to further evaluate the fluorogenicity of ***f*** **‐HM‐SiR** in the context of cellular labeling. Given the flexibility of tetrazine‐based click chemistry, we tested a variety of procedures (Figure 2 a). Protein labeling was demonstrated using the enzymatic self‐labeling HaloTag as a fusion with histone H2A or mitochondrial import receptor subunit TOM20 in combination with HaloTag ligand dienophiles. Further, we used triphenylphosphonium (TPP), a small‐molecule organelle marker, to localize the respective dienophile conjugate TPP‐BCN to the mitochondrial matrix.^19^ Staining and fluorescence imaging of nuclear and mitochondrial targets were carried out in HeLa cells. Cells were transiently transfected with H2A‐HaloTag and treated with HTL‐BCN (10 μm) for 30 min. Additional staining of unmodified HaloTag proteins with HTL‐TMR subsequent to HTL‐BCN incubation served as a transfection and co‐localization control.

After wash‐out of excess HTL‐BCN, cells were incubated with ***f*** **‐HM‐SiR** and imaged without dye removal. Averaging 2000 frames of the recorded blinking signal resulted in an image with good co‐localization to the HTL‐TMR control (Figure 2 b). In the same fashion, mitochondrial labeling with (E)‐cyclooct‐2‐en‐1‐ol (TCO^*^) as dienophile was equally successful (Supporting Information, Figure S8). Similarly, HeLa cells treated with TPP‐BCN and ***f*** **‐HM‐SiR** show expected mitochondrial structures (Figure 2 c). Co‐staining with TOM20‐HaloTag and HTL‐TMR verifies the specificity of the mitochondrial staining at good contrast. This demonstrates the efficient background suppression when using ***f*** **‐HM‐SiR** in live‐cell fluorescence microscopy. In control experiments without HTL‐BCN we noted slight unspecific signal upon addition of ***f*** **‐HM‐SiR** (Figure 2 b). We attribute this to the presence of lysosomes with luminal pH≈4. In this context, we validated the previously observed fluorogenicity of ***f*** **‐HM‐SiR** in comparison to other silicon rhodamine dyes like **HM‐SiR** (lacking a tetrazine moiety), tetrazine‐substituted **SiR‐Tz**, and bare **SiR‐COOH** (Supporting Information, Figure S6). To this end, we incubated wild type HeLa cells with the respective probes and quantified cellular fluorescence intensities (Figure 2 d,e). The average fluorescence from HeLa cells incubated with ***f*** **‐HM‐SiR** was comparable to untreated cells, while the other three SiR dyes caused significantly higher signal. The observed signal intensities directly correlate to the residual background resulting from nonreacted dye excess in labeling experiments. These tendencies were even more pronounced under no‐wash conditions (Supporting Information, Figure S9). This highlights the improved background suppression of ***f*** **‐HM‐SiR** compared to existing SiR derivatives owing to the close proximity of tetrazine to the chromophoric center.

**Figure 3:**
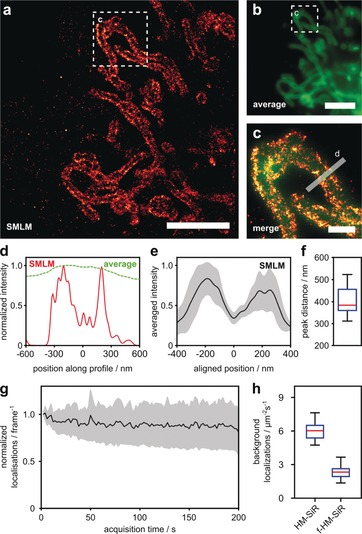
Spontaneous blinking of ***f*** **‐HM‐SiR** enables SMLM with improved resolution and reduced background. a) COS‐7 cells transiently expressing TOM20‐mCherry‐HaloTag were incubated with HTL‐TCO^*^ (10 μm), washed, and labeled with ***f*** **‐HM‐SiR** (2 μm) for SMLM imaging. See Movie S1 in the Supporting Information. b) Average projection of 500 raw data frames used for reconstruction in (a). c) Merged zoom‐ins of boxed regions in (a) (red) and (b) (green). d) Line profile along boxed region in (c) comparing normalized intensities in averaged raw data (green) and SMLM reconstruction (red). e) Averaged cross‐sectional profiles of labeled mitochondria ±1 standard deviation, *n*=16. Individual profiles were aligned to minimum between peaks (see methods and Figure S11 in the Supporting Information for all profiles). f) Peak‐to‐peak distances in cross‐sectional profiles shown in (e). g) Long‐term stability of ***f*** **‐HM‐SiR** labeled TOM20‐HaloTag in fixed COS‐7 cells. Mean localizations per frame normalized to number of localizations in first frame (black line) ±1 standard deviation, *n*=8. h) Background localization rate for HM‐SiR and ***f*** **‐HM‐SiR** in non‐transfected HeLa cells, *n*=20. Scale bars: a,b) 5 μm, c) 1 μm.

Unspecific signal reduces contrast and structure representation quality in fluorescence imaging. In SMLM it additionally leads to decreased localization precision. Therefore, efficient background suppression is particularly important here. Thus, small‐molecule probes bearing bioorthogonal attachment functionalities that come along with fluorogenic properties possess great potential for super‐resolution microscopy. In the past, super‐resolution microscopy with fluorogenic tetrazine dyes has been achieved in fixed cells.16g, 16h Encouraged by the advantageous features of ***f*** **‐HM‐SiR** in live‐cell labeling, we studied its suitability for SMLM in living cells. HeLa cells transiently transfected with TOM20‐HaloTag were incubated with HaloTag ligand‐TCO^*^ (HTL‐TCO^*^), washed and labeled with ***f*** **‐HM‐SiR** (2 μm). To minimize background signal for live‐cell SMLM excess fluorophore was washed out in this experiment. Imaging under highly inclined illumination conditions allowed the localization of individual spontaneously blinking emitters. The image reconstructed from 500 frames shows the expected structure for outer mitochondrial membrane localized TOM20 (Figure 3 a) and enabled us to follow dynamics at sub‐diffraction resolution (Supporting Information, Movie S1). Comparison with an image averaged over 500 frames in Figure 3 b highlights the achieved resolution improvement of ***f*** **‐HM‐SiR** in live‐cell SMLM. From the reconstructed image, it is possible to differentiate the outer membrane of the mitochondrial network (Figure 3 b,d) and we were able to determine a mean mitochondrial diameter of about 375 nm from 16 cross‐sectional profiles (Figure 3 e,f and Supporting Information, Figure S11). SMLM image sequences were recorded at a laser intensity of 1 kW cm^−2^ at 640 nm, at which we did not observe any considerable phototoxicity, even in long time experiments (Supporting Information, Movie S5). As these moderate laser intensities mitigate photobleaching, we were interested in the capability of ***f*** **‐HM‐SiR** for long‐term SMLM. In order to genuinely quantify the number of localizations over time unbiased by cellular movement, we chemically fixed the cells after addition of the probe. The average number of localizations sampled over 200 s showed no significant decrease (Figure 3 g). At the same time, super‐resolution images reconstructed from frame subsets do not indicate any noticeable loss in reconstruction quality. Finally, we tested how the fluorogenicity of ***f*** **‐HM‐SiR** affects the localization of unspecifically bound label in comparison to the non‐fluorogenic **HM‐SiR**. Comparison of the background localizations within a cell per area and time unit shows a more than 2‐fold reduction for ***f*** **‐HM‐SiR** (Figure 3 h). Together, these observations suggest a high suitability of ***f*** **‐HM‐SiR** to probe cellular dynamics in live‐cell SMLM.

**Figure 4:**
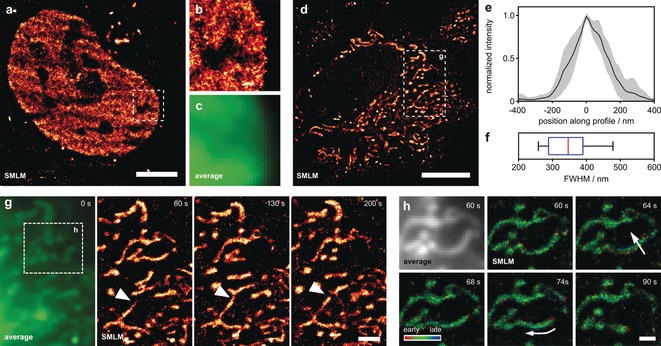
***f*** **‐HM‐SiR** reveals cellular dynamics in live HeLa cells with improved resolution. a) HeLa cells transiently expressing H2A‐HaloTag were incubated with HTL‐BCN (10 μm), washed and labeled with ***f*** **‐HM‐SiR** (2 μm). A reconstruction from 333 frames corresponding to 6.67 seconds acquisition time is shown. b) Zoom‐in of boxed region in (a). c) Corresponding averaged image for boxed region in (a). d) HeLa cells were incubated with TPP‐BCN (10 μm), washed, and labeled with ***f*** **‐HM‐SiR** (2 μm). Reconstruction from 500 frames (10 seconds) is shown. e) Averaged cross‐sectional profiles from mitochondrial tubules after alignment, ±1 standard deviation, *n*=11. f) Width of individual profiles shown in (e). See Figure S11 in the Supporting Information for position off all profiles. g) Zoom‐in of boxed region in (d). Average (left) and reconstructions from 500 frames at different time points (right). Arrowhead indicates mitochondrial fusion event. h) Average (top left) and reconstructions of boxed region in (g). Localizations are colored with respect to their relative time of appearance within a single reconstruction. Scale bars: a) 5 μm, d) 10 μm, g) 2 μm, h) 1 μm.

To fully make use of excitation‐independent on–off switching of ***f*** **‐HM‐SiR**, we performed long‐term SMLM experiments, this time omitting unnecessary dye wash‐out in order to fully utilize the probe's fluorogenicity. First, we turned our attention to the protein H2A‐HaloTag, localized in the nucleus. After incubating HeLa cells with HTL‐BCN and subsequent linker wash‐out, cells were labeled with ***f*** **‐HM‐SiR**. Mere buffer medium change and direct image acquisition was sufficient to afford high resolution in the reconstructed image (Figure 4 a). In contrast to an average projection (Figure 4 b,c), only the super‐resolved image allows for an identification of individual H2A proteins and variations in local H2A density. Subsequently, to visualize a more dynamic cellular target we localized the dienophile to the mitochondrial matrix with TPP. HeLa cells were incubated with TPP‐BCN, washed and stained with ***f*** **‐HM‐SiR**. Medium was replaced once to minimize extracellular deposition of the probe before imaging the cells. We choose a moderate excitation power of 1 kW cm^−2^ to facilitate long time imaging. As TPP accumulates in the mitochondrial matrix, the reconstructed image in Figure 4 d shows the expected pattern of homogeneously filled tubes. The averaged intersection from eleven cross‐sectional profiles (Figure 4 e) has a median FWHM of 346 nm (Figure 4 f), which is in accordance with the median of the peak‐to‐peak distance of 375 nm determined for mitochondria labeled at the outer membrane (See Figure 3 e,f and Figure S11 c in the Supporting Information for a direct comparison).

We were also able to follow mitochondrial dynamics over 200 s with a temporal resolution of 10 s as shown in Figure 4 g and Movie S2 in the Supporting Information. Considering the inherent trade‐off between high excitation power (resulting in high photon output and thus localization precision) and cell viability, the achieved spatial resolution is reasonable (for photon count and localization uncertainty see Figure S10 in the Supporting Information). The time‐resolved super‐resolution imaging was not limited by fluorophore bleaching, emphasizing the performance of ***f*** **‐HM‐SiR**. Beyond visualizing motion dynamics, it was also possible to observe mitochondrial fusion and fission events (Figure 4 g). Temporal resolution in SMLM depends on the time during which emitter localizations are accumulated for reconstructing a super‐resolved image. Increased time resolution can be achieved by shortening the accumulation time at the cost of reconstruction quality, that is, the captured localization density along the imaged structures.

To capture fast cellular dynamics, we additionally made use of the exact time at which individual localizations were recorded. We visualized this additional information by color coding the appearance time of localizations within each reconstruction from red (beginning) over green to blue (end) of each reconstruction window (Figure 4 h and Supporting Information, Movie S3). In this representation, structures which remain quasi stationary during data recording for one reconstruction will appear green while forming structures will be colored red and collapsing structure will be colored blue (Figure 4 h, arrows). The fact that most mitochondria in the shown example are colored green further indicates that the achieved time resolution of 10 s is sufficient to capture the observed mitochondrial dynamics.

Overall, these results show that the combination of fluorogenicity and self‐blinking in ***f*** **‐HM‐SiR** greatly improves the potential of SMLM applications for live‐cell experiments. While fluorogenicity significantly reduces artifacts from unspecific probe deposition, self‐blinking enables live‐cell SMLM over relatively long time scales due to the low excitation power required.

## Conclusion

In summary, we present the first combination of fluorogenic and self‐blinking properties in a single fluorescent probe and demonstrate its advantage for live‐cell localization microscopy. The tetrazine moiety strongly quenches the fluorescence prior to reaction and enables fluorogenic bioconjugation to various dienophile‐modified cellular targets. ***f*** **‐HM‐SiR** exhibits excellent cell permeability, high brightness, and photostability, all of which are important criteria for application in live‐cell imaging. Exploiting the self‐blinking properties, we demonstrate its application in live‐cell SMLM without the necessity of stabilizing buffers or high excitation power. ***f*** **‐HM‐SiR** allows live‐cell localization microscopy after simple media replacement. SMLM imaging of intracellular dynamics can be performed over long time periods and at a high spatiotemporal resolution. We expect that our molecular design will stimulate the future development of multifunctional fluorescent probes as tailor‐made tools for bioimaging.

## Conflict of interest

The authors declare no conflict of interest.

## Supporting information

As a service to our authors and readers, this journal provides supporting information supplied by the authors. Such materials are peer reviewed and may be re‐organized for online delivery, but are not copy‐edited or typeset. Technical support issues arising from supporting information (other than missing files) should be addressed to the authors.

Supplementary

Supplementary

Supplementary

Supplementary

Supplementary

Supplementary

## References

[1] L. D. Lavis , Biochemistry 2017, 56, 5165–5170. 28704030 10.1021/acs.biochem.7b00529

[2] M. Sauer , M. Heilemann , Chem. Rev. 2017, 117, 7478–7509. 28287710 10.1021/acs.chemrev.6b00667

[3a] S. Wäldchen , J. Lehmann , T. Klein , S. van de Linde , M. Sauer , Sci. Rep. 2015, 5, 15348; 26481189 10.1038/srep15348 PMC4611486

[3b] P. P. Laissue , R. A. Alghamdi , P. Tomancak , E. G. Reynaud , H. Shroff , Nat. Methods 2017, 14, 657. 28661494 10.1038/nmeth.4344

[4a] S.-n. Uno , M. Kamiya , T. Yoshihara , K. Sugawara , K. Okabe , M. C. Tarhan , H. Fujita , T. Funatsu , Y. Okada , S. Tobita , Y. Urano , Nat. Chem. 2014, 6, 681; 25054937 10.1038/nchem.2002

[4b] S.-n. Uno , M. Kamiya , A. Morozumi , Y. Urano , Chem. Commun. 2018, 54, 102–105; 10.1039/c7cc07783a 29214255

[4c] M. Schwering , A. Kiel , A. Kurz , K. Lymperopoulos , A. Sprödefeld , R. Krämer , D.-P. Herten , Angew. Chem. Int. Ed. 2011, 50, 2940–2945; 10.1002/anie.201006013 21404374

[5a] Y. Koide , Y. Urano , K. Hanaoka , T. Terai , T. Nagano , ACS Chem. Biol. 2011, 6, 600–608; 21375253 10.1021/cb1002416

[5b] G. Lukinavičius , K. Umezawa , N. Olivier , A. Honigmann , G. Yang , T. Plass , V. Mueller , L. Reymond , I. R. Corrêa, Jr. , Z.-G. Luo , C. Schultz , E. A. Lemke , P. Heppenstall , C. Eggeling , S. Manley , K. Johnsson , Nat. Chem. 2013, 5, 132. 23344448 10.1038/nchem.1546

[6] S. van de Linde , S. Wolter , M. Heilemann , M. Sauer , J. Biotechnol. 2010, 149, 260–266. 20176060 10.1016/j.jbiotec.2010.02.010

[7a] H. Takakura , Y. Zhang , R. S. Erdmann , A. D. Thompson , Y. Lin , B. McNellis , F. Rivera-Molina , S.-n. Uno , M. Kamiya , Y. Urano , J. E. Rothman , J. Bewersdorf , A. Schepartz , D. Toomre , Nat. Biotechnol. 2017, 35, 773; 28671662 10.1038/nbt.3876 PMC5609855

[7b] A. D. Thompson , J. Bewersdorf , D. Toomre , A. Schepartz , Biochemistry 2017, 56, 5194–5201. 28792749 10.1021/acs.biochem.7b00545 PMC5854879

[8] B. L. Oliveira , Z. Guo , G. J. L. Bernardes , Chem. Soc. Rev. 2017, 46, 4895–4950. 28660957 10.1039/c7cs00184c

[9] H. E. Murrey , J. C. Judkins , C. W. am Ende , T. E. Ballard , Y. Fang , K. Riccardi , L. Di , E. R. Guilmette , J. W. Schwartz , J. M. Fox , D. S. Johnson , J. Am. Chem. Soc. 2015, 137, 11461–11475. 26270632 10.1021/jacs.5b06847 PMC4572613

[10a] D. S. Liu , A. Tangpeerachaikul , R. Selvaraj , M. T. Taylor , J. M. Fox , A. Y. Ting , J. Am. Chem. Soc. 2012, 134, 792–795; 22176354 10.1021/ja209325n PMC3381951

[10b] M. Best , A. Degen , M. Baalmann , T. T. Schmidt , R. Wombacher , ChemBioChem 2015, 16, 1158–1162; 25900689 10.1002/cbic.201500042

[10c] M. Baalmann , M. J. Ziegler , P. Werther , J. Wilhelm , R. Wombacher , Bioconjugate Chem. 2019, 30, 1405–1414. 10.1021/acs.bioconjchem.9b00157 30883100

[11a] J. L. Seitchik , J. C. Peeler , M. T. Taylor , M. L. Blackman , T. W. Rhoads , R. B. Cooley , C. Refakis , J. M. Fox , R. A. Mehl , J. Am. Chem. Soc. 2012, 134, 2898–2901; 22283158 10.1021/ja2109745 PMC3369569

[11b] K. Lang , L. Davis , J. Torres-Kolbus , C. Chou , A. Deiters , J. W. Chin , Nat. Chem. 2012, 4, 298; 22437715 10.1038/nchem.1250 PMC3758886

[11c] K. Lang , L. Davis , S. Wallace , M. Mahesh , D. J. Cox , M. L. Blackman , J. M. Fox , J. W. Chin , J. Am. Chem. Soc. 2012, 134, 10317–10320; 22694658 10.1021/ja302832g PMC3687367

[11d] T. Plass , S. Milles , C. Koehler , J. Szymański , R. Mueller , M. Wießler , C. Schultz , E. A. Lemke , Angew. Chem. Int. Ed. 2012, 51, 4166–4170; 10.1002/anie.201108231 22473599

[12a] J. Schoch , M. Wiessler , A. Jäschke , J. Am. Chem. Soc. 2010, 132, 8846–8847; 20550120 10.1021/ja102871p

[12b] J. Schoch , S. Ameta , A. Jäschke , Chem. Commun. 2011, 47, 12536–12537; 10.1039/c1cc15476a 22002170

[12c] J. Schoch , M. Staudt , A. Samanta , M. Wiessler , A. Jäschke , Bioconjugate Chem. 2012, 23, 1382–1386; 10.1021/bc300181n 22709568

[12d] H. Bußkamp , E. Batroff , A. Niederwieser , O. S. Abdel-Rahman , R. F. Winter , V. Wittmann , A. Marx , Chem. Commun. 2014, 50, 10827–10829; 10.1039/c4cc04332d 25089682

[12e] U. Rieder , N. W. Luedtke , Angew. Chem. Int. Ed. 2014, 53, 9168–9172; 10.1002/anie.201403580 24981416

[13a] J. Yang , Y. Liang , J. Šečkutė , K. N. Houk , N. K. Devaraj , Chem. Eur. J. 2014, 20, 3365–3375; 24615990 10.1002/chem.201304225 PMC4020353

[13b] A. Niederwieser , A.-K. Späte , L. D. Nguyen , C. Jüngst , W. Reutter , V. Wittmann , Angew. Chem. Int. Ed. 2013, 52, 4265–4268; 10.1002/anie.201208991 23468318

[13c] D. M. Patterson , K. A. Jones , J. A. Prescher , Mol. BioSyst. 2014, 10, 1693–1697; 24623192 10.1039/c4mb00092g

[13d] F. Doll , A. Buntz , A.-K. Späte , V. F. Schart , A. Timper , W. Schrimpf , C. R. Hauck , A. Zumbusch , V. Wittmann , Angew. Chem. Int. Ed. 2016, 55, 2262–2266; 10.1002/anie.201503183 26756572

[14] J. Yang , J. Šečkutė , C. M. Cole , N. K. Devaraj , Angew. Chem. Int. Ed. 2012, 51, 7476–7479; 10.1002/anie.201202122 PMC3431913 22696426

[15a] N. K. Devaraj , S. Hilderbrand , R. Upadhyay , R. Mazitschek , R. Weissleder , Angew. Chem. Int. Ed. 2010, 49, 2869–2872; 10.1002/anie.200906120 PMC3433403 20306505

[15b] G. Lukinavičius , L. Reymond , E. D'Este , A. Masharina , F. Göttfert , H. Ta , A. Güther , M. Fournier , S. Rizzo , H. Waldmann , C. Blaukopf , C. Sommer , D. W. Gerlich , H.-D. Arndt , S. W. Hell , K. Johnsson , Nat. Methods 2014, 11, 731; 24859753 10.1038/nmeth.2972

[15c] S. H. Alamudi , R. Satapathy , J. Kim , D. Su , H. Ren , R. Das , L. Hu , E. Alvarado-Martínez , J. Y. Lee , C. Hoppmann , E. Peña-Cabrera , H.-H. Ha , H.-S. Park , L. Wang , Y.-T. Chang , Nat. Commun. 2016, 7, 11964. 27321135 10.1038/ncomms11964 PMC4915154

[16a] J. C. T. Carlson , L. G. Meimetis , S. A. Hilderbrand , R. Weissleder , Angew. Chem. Int. Ed. 2013, 52, 6917–6920; 10.1002/anie.201301100 PMC3875324 23712730

[16b] L. G. Meimetis , J. C. T. Carlson , R. J. Giedt , R. H. Kohler , R. Weissleder , Angew. Chem. Int. Ed. 2014, 53, 7531–7534; 10.1002/anie.201403890 PMC4122131 24915832

[16c] H. Wu , J. Yang , J. Šečkutė , N. K. Devaraj , Angew. Chem. Int. Ed. 2014, 53, 5805–5809; 10.1002/anie.201400135 PMC4104127 24764312

[16d] A. Wieczorek , T. Buckup , R. Wombacher , Org. Biomol. Chem. 2014, 12, 4177–4185; 24826902 10.1039/c4ob00245h

[16e] A. Wieczorek , P. Werther , J. Euchner , R. Wombacher , Chem. Sci. 2017, 8, 1506–1510; 28572909 10.1039/c6sc03879d PMC5452268

[16f] P. Werther , J. S. Möhler , R. Wombacher , Chem. Eur. J. 2017, 23, 18216–18224; 29044851 10.1002/chem.201703607

[16g] E. Kozma , G. Estrada Girona , G. Paci , E. A. Lemke , P. Kele , Chem. Commun. 2017, 53, 6696–6699; 10.1039/c7cc02212c 28530747

[16h] G. Knorr , E. Kozma , J. M. Schaart , K. Németh , G. Török , P. Kele , Bioconjugate Chem. 2018, 29, 1312–1318; 10.1021/acs.bioconjchem.8b00061 29431990

[16i] Y. Lee , W. Cho , J. Sung , E. Kim , S. B. Park , J. Am. Chem. Soc. 2018, 140, 974–983; 29240995 10.1021/jacs.7b10433

[16j] D. Wu , D. F. O'Shea , Chem. Commun. 2017, 53, 10804–10807. 10.1039/c7cc06545k 28920988

[17a] A. Nadler , C. Schultz , Angew. Chem. Int. Ed. 2013, 52, 2408–2410; 10.1002/anie.201209733 23339134

[17b] P. Shieh , C. R. Bertozzi , Org. Biomol. Chem. 2014, 12, 9307–9320. 25315039 10.1039/c4ob01632g PMC4259128

[18] K. I. Mortensen , L. S. Churchman , J. A. Spudich , H. Flyvbjerg , Nat. Methods 2010, 7, 377. 20364147 10.1038/nmeth.1447 PMC3127582

[19] J. Zielonka , J. Joseph , A. Sikora , M. Hardy , O. Ouari , J. Vasquez-Vivar , G. Cheng , M. Lopez , B. Kalyanaraman , Chem. Rev. 2017, 117, 10043–10120. 28654243 10.1021/acs.chemrev.7b00042 PMC5611849

