# Supplementary material for: Live‐Cell Localization Microscopy with a Fluorogenic and Self‐Blinking Tetrazine Probe
Source: Angew Chem Int Ed Engl. 2019 Nov 18;59(2):804–10. doi: 10.1002/anie.201906806 (PMC6972563; doi:10.1002/anie.201906806)
Supplement: Supplementary file 1 — Supplementary [file ANIE-59-804-s001.pdf]

Angewandte Chemie
Eine Zeitschrift der Gesellschaft Deutscher Chemiker

Supporting Information

Live-Cell Localization Microscopy with a Fluorogenic and Self-Blinking Tetrazine Probe

Philipp Werther[+], Klaus Yserentant[+], Felix Braun, Nicolai Kaltwasser, Christoph Popp, Mathis Baalmann, Dirk-Peter Herten, and Richard Wombacher**

anie_201906806_sm_miscellaneous_information.pdf
anie_201906806_sm_Movie_1.avi
anie_201906806_sm_Movie_2.avi
anie_201906806_sm_Movie_3.avi
anie_201906806_sm_Movie_4.avi
anie_201906806_sm_Movie_5.avi

Outline

1 Supporting Movie Captions 1
2 General Information 1
3 Photophysical Measurements 2
4 Synthesis 5
5 In Vitro Protein Labeling 18
6 Cell Culture 19
7 Preparation of Cells for Imaging 19
8 Fluorescence Microscopy 20
9 Supporting Microscopy Figures 22
10 SMLM Reconstructions 24
11 Localization Precision Measurements 24
12 Evaluation of Acute Phototoxicity 24
13 Image Processing and Statistical Analysis 25
14 Evaluation of Non-Specific Labeling 25
15 NMR Spectra 26
16 References 38

1 Supporting Movie Captions

1.1 Supporting movie 1

COS-7 cells transiently expressing TOM20-mCherry-HaloTag were incubated with HTL-BCN (10 µM), washed and labeled with *f*-HM-SiR (2 µM) for live cell SMLM imaging. The movie shows a sequence of reconstructions with overlapping windows of 500 frames. Scale bar 5 µm.

1.2 Supporting movie 2

HeLa cells were incubated with TPP-BCN (10 µM), washed and labeled with *f*-HM-SiR (2 µM). Zoom-in of boxed region in fig. 4d. The movie shows a sequence of reconstructions with overlapping windows of 500 frames. Scale bar 5 µm.

1.3 Supporting movie 3

HeLa cells were incubated with TPP-BCN (10 µM), washed and labeled with *f*-HM-SiR (2 µM). Zoom-in of of boxed region in fig. 4g with temporal color coding to visualize fast dynamics. Localizations are colored with respect to their relative time of appearance within a single reconstruction from red over green to blue.

1.4 Supporting movie 4

Raw data used for reconstructions in fig.4d-h and supporting movie 2 showing real-time blinking (left) of *f*-HM-SiR and corresponding reconstruction sequence (right) in HeLa cells loaded with TPP-BCN and labeled with *f*-HM-SiR.

2 General Information

Chemicals were purchased from Sigma-Aldrich Co. LLC, abcr GmbH, TCI Europe N.V., Acros Organics, Alfa Aesar, Fluorochem Ltd. and were used as received. SiR-tetrazine was supplied by Spirochrome AG. NMR solvents were purchased from euriso-top SAS. Polygram Sil G/UV254 TLC plates from Macherey-Nagel GmbH & Co KG and Sigma Aldrich were used for thin layer chromatography. Normal phase column chromatography was performed using silica gel from Fluka with a pore size of 60 Å and a particle size range of 40-63 µm. Solvents were p.a. quality.

HPLC analytics and semi-preparative purifications were conducted on an Agilent 1100 series HPLC system. Phenomenex Luna 3µ and 5µ C18 reversed-phase columns were used for these purposes (Solvent A: H_2O containing 0.1% TFA; Solvent B: MeCN containing 0.1% TFA). Collected HPLC fractions were dried by lyophilization.

Mass spectrometry was performed on a Bruker microTOF-QII mass spectrometer. NMR spectra were recorded on a Varian Mercury Plus 300 MHz spectrometer or a Varian 500 MHz NMR System. Peak shifts were reported relative to solvent peaks according to Fulmer *et al*.[1]

Fluorescence measurements were performed on a JASCO Spectrofluorometer FP-6500 or a Cary Eclipse. Absorption spectra were recorded on a Cary 100 Bio UV-Visible or a Cary 500 Scan.

Kinetic measurements involving fluorescence readout were performed on a Tecan multiplate Safire 2 multimode microplate reader.

3 Photophysical Measurements

Dye samples for photophysical measurements were prepared from 10 mM stock solutions in anhydrous DMSO and diluted to various final concentrations in aqueous phosphate buffers. Extinction coefficients and quantum yields of *f*-HM-SiR and DA_{inv}-product were determined at pH values of 3.5 and 4.0, respectively, to achieve maximum amount of the open form. Extinction coefficients were determined by measuring the absorbance at four different concentrations (1-20 µM). Quantum yields were determined as described by Brouwer[2] relative to cresyl violet in methanol ($\Phi = 0.57$[2]) at an excitation wavelength of 580 nm.

For time-course fluorescence measurements a stock solution of (1R,8S,9s)-bicyclo[6.1.0]non-4-yn-9-ylmethanol (BCN) in dimethyl sulfoxide (100 mM) was prepared. Black 96-well plates were loaded with 100 µL of 10 µM solutions *f*-HM-SiR in PBS and the fluorescence intensity start value was determined. A stock solution of BCN was added to each well (final concentration: 100 µM), the measurement was started and the fluorescence intensity read out every 10 seconds. Temperature was kept constant between 31 °C and 32 °C. Dividing end and start fluorescence intensity values from time course measurements yielded the fluorescence enhancements.

Table S1. Photophysical properties of *f*-HM-SiR and DA_{inv}-Product.

	$\lambda_{abs, max}$ [nm]	$\lambda_{em, max}$ [nm]	ε_{max} [$M^{-1}cm^{-1}$]	Φ_F
f-HM-SiR	654	670	45 000	0.06
DA_{inv}-Product	653	669	185 000	0.26

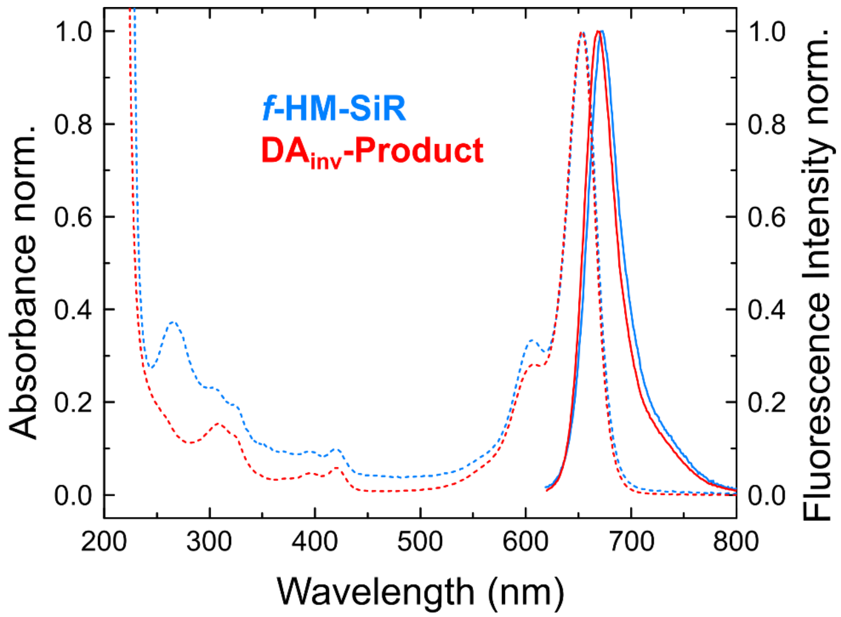

Figure S1. Absorption (dotted line) and emission spectra (straight line) of ***f*-HM-SiR** (blue, pH=3.5) and **DA_{inv}-Product** (red, pH 4.0) in PBS buffer.

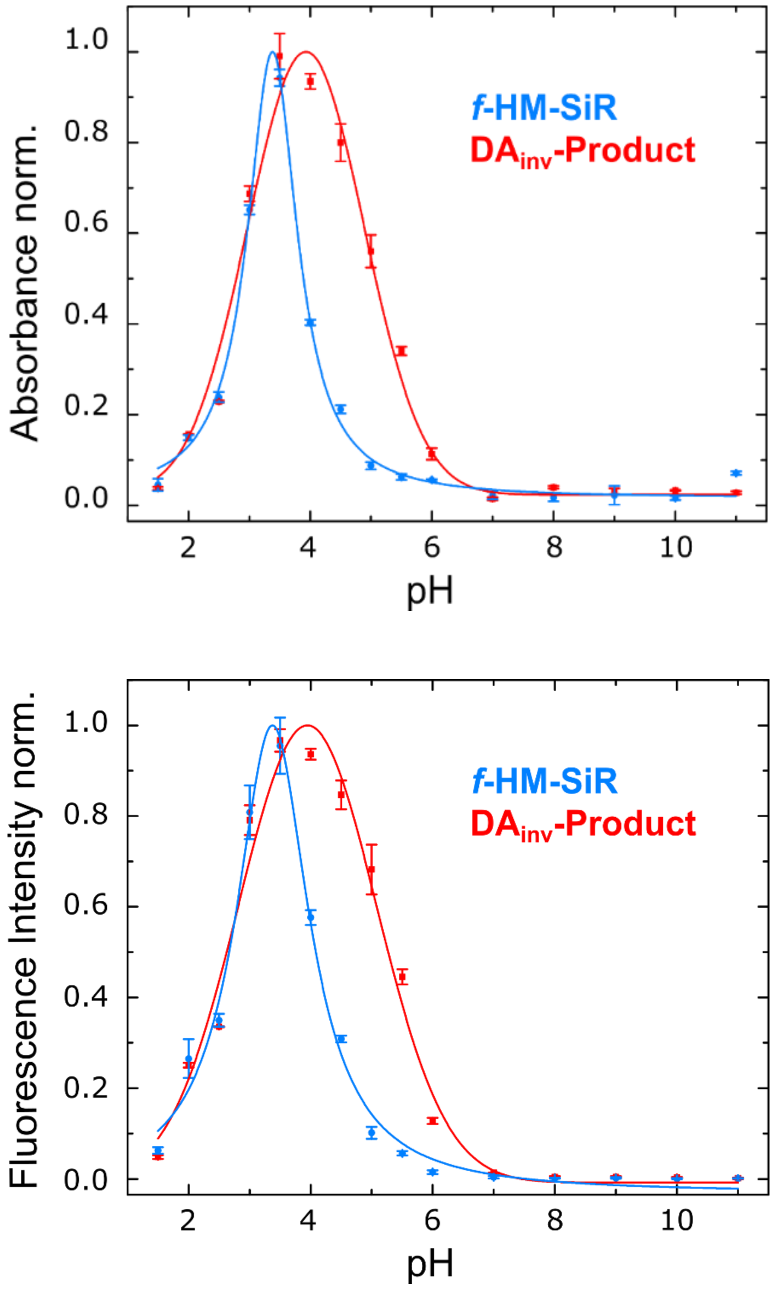

Figure S2. pH-dependent absorbance and fluorescence of ***f*-HM-SiR** and model cycloadduct **DA$_{inv}$-Product** in PBS buffer. Recorded in triplicates.

4 Synthesis

4.1 Synthesis Route

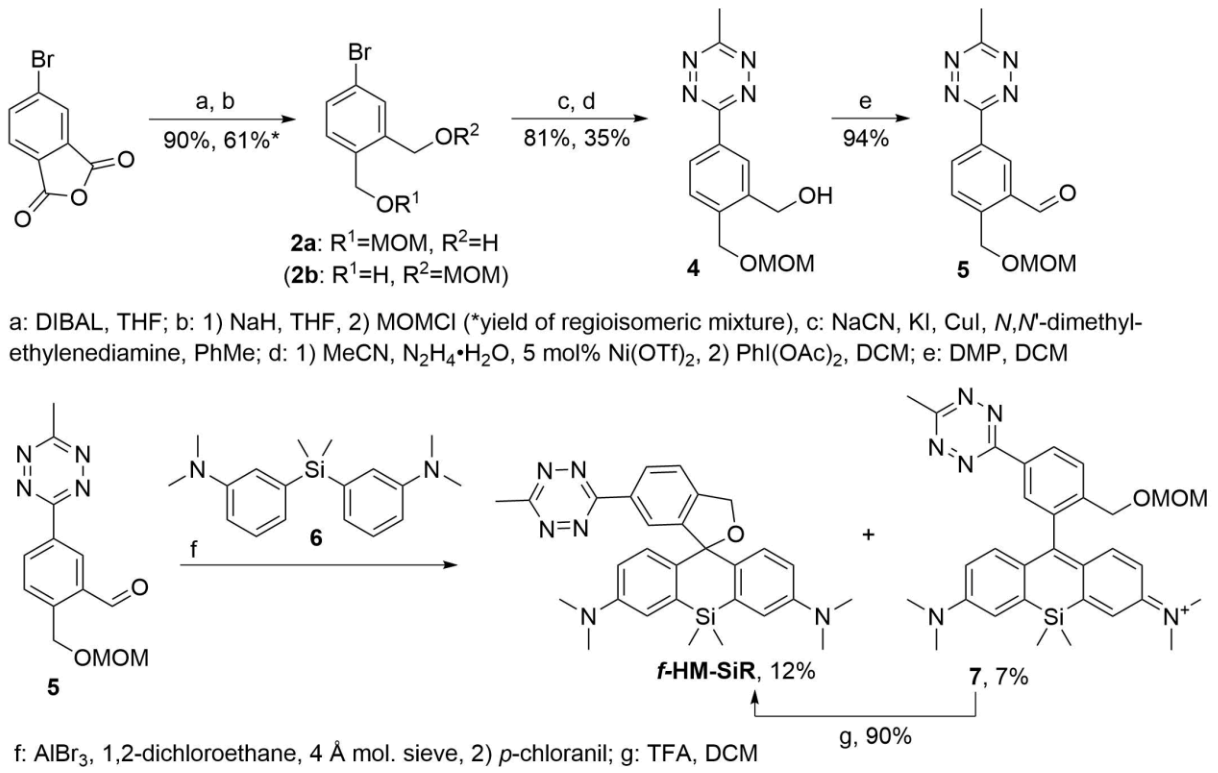

Scheme S1: a-e: Synthesis of MOM-protected tetrazinyl benzaldehyde **5**, f: Synthesis of ***f*-HM-SiR** via Friedel-Crafts reaction between **5** and diaryl silane nucleophile **6**.

4.2 Synthesis of Precursors

(4-Bromo-1,2-phenylene)dimethanol (1)

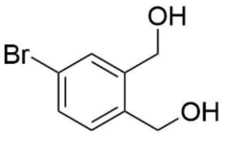

The title product was synthesized according to literature.[3] Commercially available 4-bromophthalic anhydride (7.00 g; 30.8 mmol) was dissolved in dry toluene (125 ml) under an argon atmosphere. The solution was cooled down to 0°C and DIBAL (1.76 M in dry toluene 87.6 ml, 154 mmol) was added dropwise. After complete addition, the reaction mixture was allowed to heat up to rt and stirred for 16 h. Subsequently, methanol (40 mL) and hydrochloric acid (aqueous, 1 M) was added at 0°C. The aqueous phase was separated and extracted with EtOAc. Combined organic layers were washed with brine, dried over

$MgSO_4$ and concentrated *in vacuo*. Flash chromatography (silica, EtOAc/DCM 1/1 of the solid residue afforded the title product as a white powder (6.04 g, 90%).

^{1}H NMR (300 MHz, CD_3CN) δ 7.57 (d, *J* = 2.4 Hz, 1H), 7.43 (dd, *J* = 8.1, 2.2 Hz, 1H), 7.31 (d, *J* = 8.1 Hz, 1H), 4.60 (d, *J* = 5.8 Hz, 2H), 4.57 (d, *J* = 5.7 Hz, 2H), 3.42 (t, *J* = 5.8 Hz, 1H), 3.37 (t, *J* = 5.7 Hz, 1H). HRMS (ESI$^+$) m/z 238.9678 calcd for $[C_8H_9BrNaO_2]^+$ (M+Na$^+$), 238.9683 found.

(5/4-Bromo-2-((methoxymethoxy)methyl)phenyl)methanol (2a/2b)

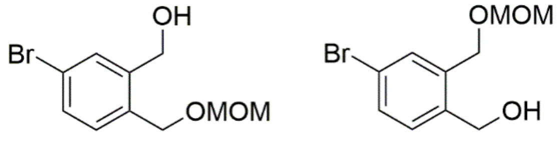

2a **2b**

2a and **2b** were synthesized based on a literature procedure.[4] NaH (60% dispersion in mineral oil, 1.89 g, 47.1 mmol) was suspended in dry THF (150 mL) under an argon atmosphere. At 0°C, **S1** (10.2 g, 47.1 mmol) was added slowly and the reaction mixture was allowed to heat up to rt and stirred overnight. At the next day, additional THF (100 mL) was added to the suspension. Chloromethyl methyl ether (3.79 g, 3.58 mL, 47.1 mmol) was dissolved in dry THF (45 ml) and slowly added to the vigorously stirred suspension. After stirring overnight, the mixture was diluted with water until formed NaCl was dissolved and extracted with EtOAc (3 x 50 ml). The combined organic layers were washed with brine, dried over $MgSO_4$ and the solvent was removed *in vacuo*. Purification by flash column chromatography (silica, 0-50% EtOAc/cyclohexane) afforded an regioisomeric mixture (7.49 g, 61%) of **2a** and **2b** as a colorless oil, along with recovered **1** (1.92 g, 19%). After multiple additional flash chromatography purification, **2a** (3.33 g, 12.8 mmol, 54%) and **2b** (3.00 g, 11.5 mmol, 49%) were obtained as colorless oils (half of the equivalent of starting material defined as 100%).

2a: ^{1}H NMR (300 MHz, $CDCl_3$) δ 7.59 (d, *J* = 2.1 Hz, 1H), 7.43 (dd, *J* = 8.1, 2.1 Hz, 1H), 7.24 (d, *J* = 8.1 Hz, 1H), 4.69 (s, 2H), 4.67 (d, *J* = 5.8 Hz, 2H), 4.64 (s, 2H), 3.40 (s, 3H), 2.64 (t, *J* = 6.2 Hz, 1H). ^{13}C NMR (75 MHz, $CDCl_3$) δ 142.2, 134.6, 132.2, 131.6, 131.1, 122.7, 95.6, 67.2, 62.9, 55.9. HRMS (ESI$^+$) m/z 282.9940 calcd for $[C_{10}H_{13}BrNaO_3]^+$ (M+Na$^+$), 282.9947 found.

2b: ^{1}H NMR (300 MHz, $CDCl_3$) δ 7.54 (d, *J* = 2.1 Hz, 1H), 7.46 (dd, *J* = 8.1, 2.1 Hz, 1H), 7.29 (d, *J* = 8.1 Hz, 1H), 4.71 (s, 2H), 4.69 – 4.61 (m, 4H), 3.41 (s, 3H), 2.56 (t, *J* = 6.2 Hz,

1H). ^{13}C NMR (75 MHz, $CDCl_3$) δ 138.8, 138.0, 132.6, 131.7, 131.1, 122.0, 96.0, 67.1, 62.9, 55.9. HRMS (ESI^+) m/z 282.9940 calcd for $[C_{10}H_{13}BrNaO_3]^+$ ($M+Na^+$), 282.9944 found.

3-(Hydroxymethyl)-4-((methoxymethoxy)methyl)benzonitrile (3)

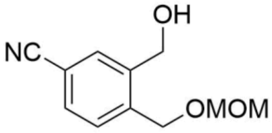

This procedure was adapted from literature.[5] Dry toluene (20 ml), *N*,*N'*-dimethylethylenediamine (945 mg, 1.15 ml, 10.7 mmol) and **2a** (2.80 g, 10.7 mmol) were degassed by freeze-pump-thawing. A microwave tube was charged with CuI (204 mg, 1.07 mmol), KI (356 mg, 2.14 mmol) and NaCN (631 mg, 12.9 mmol), sealed and backfilled with argon. Subsequently, the degassed solution was added and the reaction mixture was stirred overnight at 110°C. After cooling down to rt, 25% aqueous NH_3 solution (60 ml) was added and the mixture was stirred for 30 min. The aqueous phase was separated and extracted with EtOAc (3 x 10 ml). The combined organic layers were dried over $MgSO_4$ and the solvent was removed *in vacuo*. Purification by flash chromatography (silica, 5-50% EtOAc/cyclohexane) afforded the title product (1.79 g, 81%) as a colorless oil along with recovered **2a** (256 mg, 9%).

^{1}H NMR (300 MHz, Chloroform-*d*) δ 7.75 (d, *J* = 1.6 Hz, 1H), 7.60 (dd, *J* = 7.9, 1.7 Hz, 1H), 7.52 (d, *J* = 7.9 Hz, 1H), 4.74 (d, *J* = 6.1 Hz, 2H), 4.72 (s, 2H), 4.71 (s, 2H), 3.40 (s, 3H), 2.42 (t, *J* = 6.1 Hz, 1H). ^{13}C NMR (75 MHz, $CDCl_3$) δ 141.0, 140.6, 132.0, 130.6, 128.7, 127.6, 96.1, 67.4, 63.5, 55.9. HRMS (ESI^+) m/z 230.0788 calcd for $[C_{11}H_{13}NNaO_3]^+$ ($M+Na^+$), 230.0801 found.

(2-((Methoxymethoxy)methyl)-5-(6-methyl-1,2,4,5-tetrazin-3-yl)phenyl)-methanol (4)

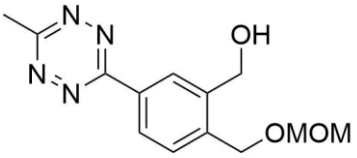

The procedure for the preparation of 1,2,4,5-tetrazines was adapted from the literature.[6]
S2 (1.79 g, 8.63 mmol), acetonitrile (3.54 g, 4.51 mL, 86.3 mmol), $Ni(OTf)_2$ (1.54 g, 4.31 mmol) and hydrazine monohydrate (21.6 g, 21.0 mL, 0.431 mol) were combined in a microwave tube. The tube was sealed and the mixture stirred at 60°C for 20 h. After cooling down to rt, the mixture was extracted with dichloromethane (3 x 100 mL). The combined

organic layers were washed with brine, dried over $MgSO_4$ and the solvent was removed *in vacuo*. The crude residue was dissolved in DCM (40 mL) and $PhI(OAc)_2$ (4.17 g, 12.9 mmol) was added. The mixture was stirred for 16 h at rt and subsequent purification by flash chromatography (silica, 10-50% EtOAc/cyclohexane), afforded the title product as pink solid (842 mg, 35%).

^{1}H NMR (300 MHz, $CDCl_3$) δ 8.66 (d, *J* = 1.7 Hz, 1H), 8.54 (dd, *J* = 8.0, 1.9 Hz, 1H), 7.63 (d, *J* = 7.9 Hz, 1H), 4.84 (d, *J* = 5.6 Hz, 2H), 4.81 (s, 2H), 4.76 (s, 2H), 3.44 (s, 3H), 3.11 (s, 3H), 2.64 (t, *J* = 6.2 Hz, 1H). ^{13}C NMR (75 MHz, $CDCl_3$) δ 167.5, 163.9, 140.9, 140.6, 132.0, 130.6, 128.7, 127.6, 96.1, 67.4, 63.4, 55.9, 21.3. HRMS (ESI$^+$) m/z 299.1115 calcd for $[C_{13}H_{16}N_4NaO_3]^+$ (M+Na$^+$), 299.1121 found.

2-((Methoxymethoxy)methyl)-5-(6-methyl-1,2,4,5-tetrazin-3-yl)benzaldehyde (5)

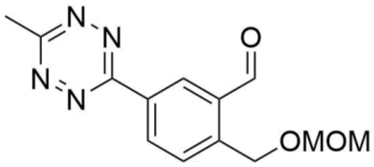

4 (993 mg, 3.59 mmol) was dissolved in DCM (36 ml) and Dess-Martin periodinane (1.68 g, 3.95 mmol) was added. The mixture was stirred for 1 h at rt until TLC monitoring indicated complete conversion. Subsequently the solvent was evaporated and the mixture purified by flash chromatography (silica, 5-20% EtOAc/cyclohexane), yielding the title product (927 mg, 94%) as pink solid.

^{1}H NMR (300 MHz, $CDCl_3$) δ 10.32 (s, 1H), 9.09 (d, *J* = 1.9 Hz, 1H), 8.83 (dd, *J* = 8.1, 1.9 Hz, 1H), 7.98 (d, *J* = 8.1 Hz, 1H), 5.15 (s, 2H), 4.83 (s, 2H), 3.44 (s, 3H), 3.14 (s, 3H). ^{13}C NMR (75 MHz, $CDCl_3$) δ 192.4, 167.9, 163.3, 145.3, 134.1, 132.8, 132.7, 131.6, 129.0, 96.6, 66.9, 55.8, 21.4. HRMS (ESI$^+$) m/z 297.0958 calcd for $[C_{13}H_{14}N_4NaO_3]^+$ (M+Na$^+$), 297.0963 found.

3-Hydroxymethyl-6-(6-methyl-1,2,4,5-tetrazin-3-yl)*N,N,N'*,*N'*-tetramethyl-Si-rhodamine (*f*-HM-SiR)

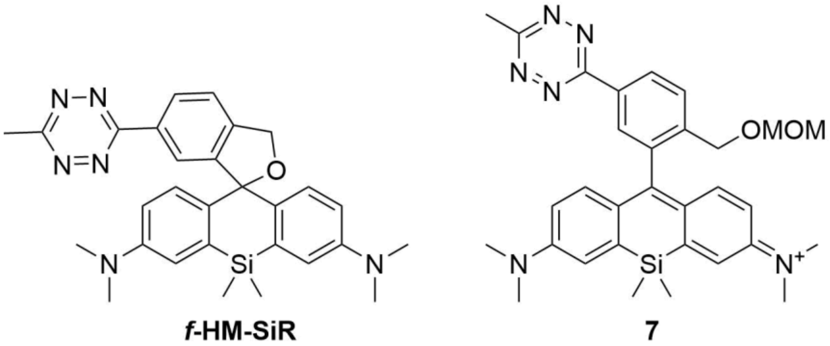

3,3'-(Dimethylsilanediyl)bis(*N*,*N*-dimethylaniline) **6** (190 mg, 638 µmol) and $AlBr_3$ solution (1 M in dry dibromomethane, 638 µmol) were added to a Schlenk tube with activated molecular sieves (4 Å) under argon. Aldehyde **3** (350 mg, 1.28 mmol) was dissolved in 1,2-dichlorethane (5.5 ml) and added to the suspension. After stirring for 20 h at room temperature, *p*-chloranil (157 mg, 638 µmol) was added and the reaction mixture was stirred for another 2 h. Purification by flash chromatograph (0-10% MeOH/DCM with 1% acetic acid) afforded ***f*-HM-SiR** (39 mg, 12%) as a slightly pink to deep blue solid along with **7** (29 mg, 7.2%[1]) as a blue solid.

7 (29 mg, 46 µmol) was further dissolved in dry DCM (0.3 ml) and trifluoroacetic acid (175 µL, 2.29 µmol) was added dropwise at 0°C. Upon addition of the acid, the solution turned from deep blue to yellow. The cooling bath was removed and the mixture stirred for 3 h at rt. The solvent was removed *in vacuo* and purification by flash chromatography (silica, 0-10% MeOH/DCM with 1% acetic acid) yielded ***f*-HM-SiR** (21 mg, 90%).

Combined yield of ***f*-HM-SiR**: (60 mg, 18%).

Prior to photophysical or cell experiments, HPLC purification was performed (20-90% Solvent B/Solvent A).

***f*-HM-SiR (open form):** ^{1}H NMR (500 MHz, CD_3CN) δ 8.71 (d, *J* = 7.7 Hz, 1H), 8.27 (s, 1H), 7.96 (d, *J* = 8.1 Hz, 1H), 7.30 (d, *J* = 1.9 Hz, 2H), 7.11 (d, *J* = 9.5 Hz, 2H), 6.68 (dd, *J* = 9.5, 1.8 Hz, 2H), 4.38 (s, 2H), 3.28 (s, 12H), 3.02 (s, 3H), 0.60 (s, 6H). ^{13}C NMR (126 MHz, CD_3CN) δ 168.7, 167.4, 164.6, 155.2, 149.1, 145.2, 141.8, 139.1, 132.2, 129.0, 128.9, 128.9, 128.1, 122.2, 115.1, 61.9, 41.3, 21.4, -1.0, -1.1. ***f*-HM-SiR (spiroether form):** ^{1}H NMR (500

---

1 Bromide was assumed as counter ion for yield determination.

MHz, CD_3CN) δ 8.35 (dd, *J* = 8.0, 1.5 Hz, 1H), 7.88 (dd, *J* = 1.5, 0.6 Hz, 1H), 7.57 (dd, *J* = 8.0, 0.8 Hz, 1H), 7.28 (d, *J* = 8.9 Hz, 2H), 6.99 (d, *J* = 3.0 Hz, 2H), 6.73 (dd, *J* = 8.9, 2.9 Hz, 2H), 5.64 (s, 2H), 2.92 (s, 3H), 2.91 (s, 12H), 0.72 (s, 3H), 0.52 (s, 3H). ^{13}C NMR (126 MHz, CD_3CN) δ 168.3, 164.5, 151.6, 150.1, 142.5, 140.2, 134.0, 133.0, 129.1, 127.3, 123.9, 122.6, 117.2, 115.5, 93.2, 75.0, 40.6, 21.3, 0.6, 0.0.
HRMS (ESI^+) m/z 509.2480 calcd for $[C_{29}H_{33}N_6OSi]^+$ ($M+H^+$), 509.2484 found.

7: ^1H NMR (500 MHz, CD_3CN) δ 8.70 (dd, *J* = 8.2, 1.8 Hz, 1H), 8.31 (d, *J* = 1.8 Hz, 1H), 7.93 (d, *J* = 8.2 Hz, 1H), 7.32 (d, *J* = 2.9 Hz, 2H), 7.09 (d, *J* = 9.7 Hz, 2H), 6.68 (dd, *J* = 9.7, 2.9 Hz, 2H), 4.47 (s, 2H), 4.36 (s, 2H), 3.28 (s, 12H), 3.10 (s, 3H), 3.01 (s, 3H), 0.62 (s, 3H), 0.60 (s, 3H). ^{13}C NMR (126 MHz, CD_3CN) δ 168.8, 166.9, 164.5, 155.2, 149.0, 141.9, 141.8, 140.0, 132.8, 130.5, 129.1, 128.9, 128.2, 122.3, 115.1, 97.0, 67.2, 55.6, 41.4, 21.5, 1.3, -0.9, -1.0. HRMS (ESI^+) m/z 553.2742 calcd for $[C_{31}H_{37}N_6O_2Si]^+$ ($M+H^+$), 553.2749 found.

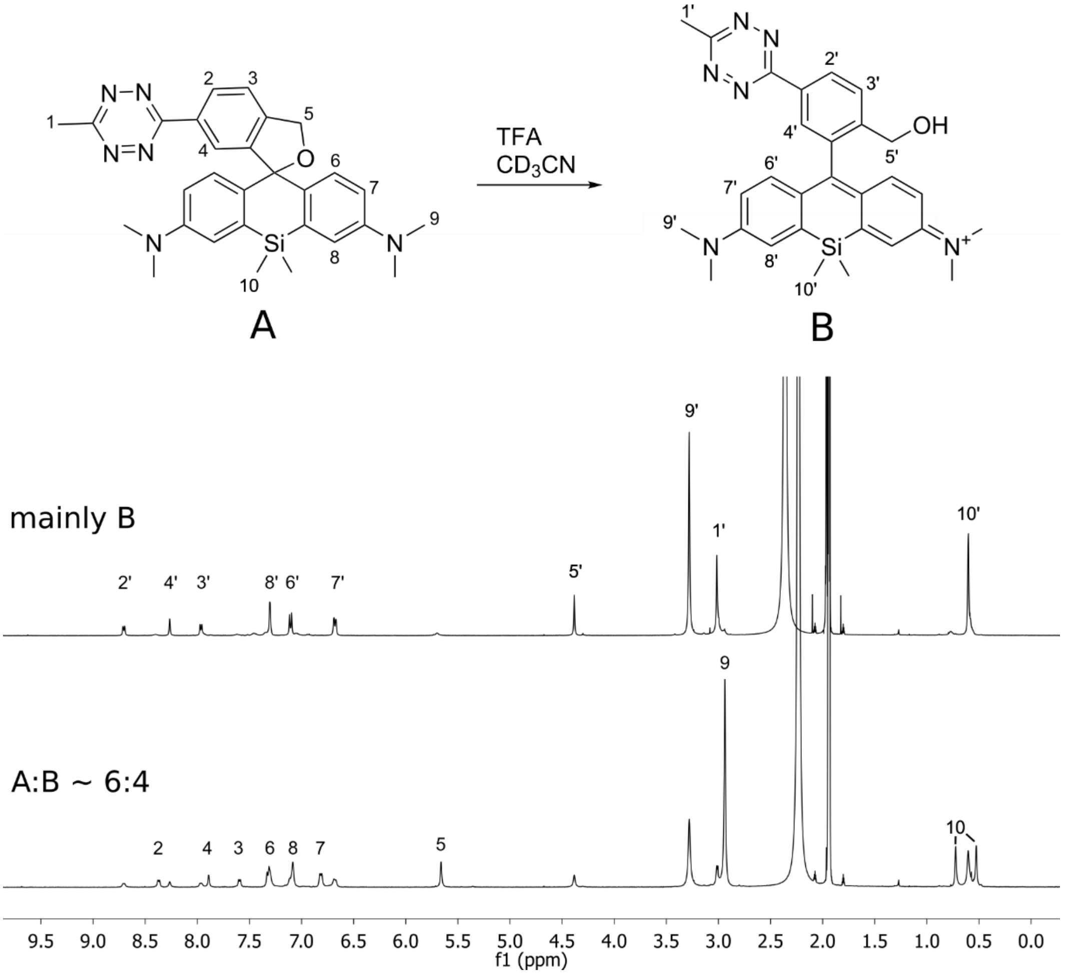

Figure S3. ^1H NMR spectra (500 MHz, CD_3CN) of ***f*-HM-SiR** before (below) and after (up) the addition of TFA enables to distinguish between spiroether (A) and hydroxyl isomer (B).

DA_{inv}-Product

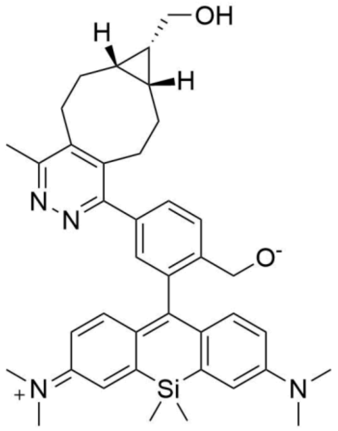

((1R,8S,9s)-Bicyclo[6.1.0]non-4-yn-9-yl)methanol **BCN** (4.1 mg, 28 µmol) was dissolved in MeCN (0.5 mL) and ***f*-HM-SiR** (70 µL, 0.1 mg/µL in MeCN, 14 µmol) was added. The solution was stirred overnight at rt and purified by HPLC (20-90% Solvent B/Solvent A).

HRMS (ESI^+) m/z 631.3463 calcd for [$C_{39}H_{47}N_4O_2Si^+$] ($M+H^+$), 631.3467 found; m/z 316.1768 calcd for [$C_{39}H_{48}N_4O_2Si^{2+}$] ($M+2H^+$), 316.1775 found.

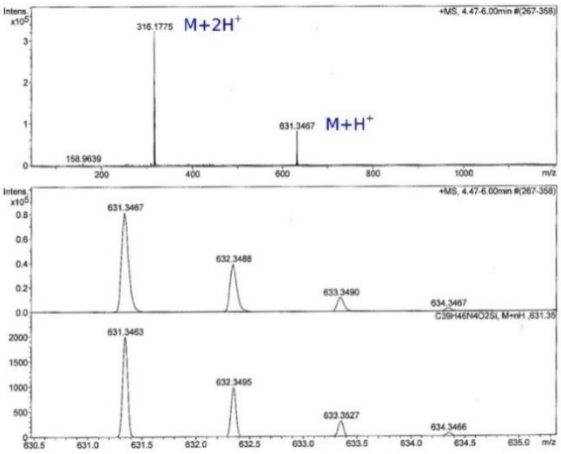

Figure S4. HRMS (ESI^+) of DA_{inv}-Product.

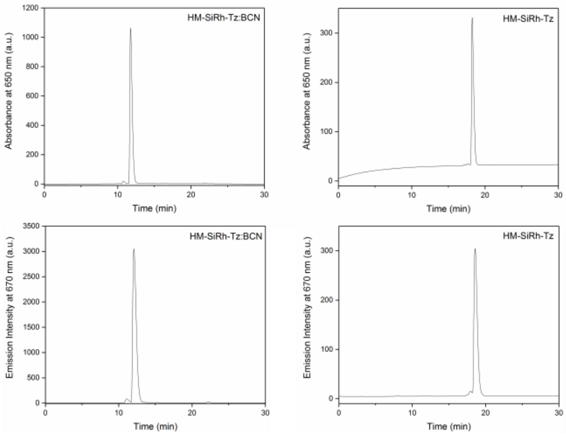

Figure S5. Analytical HPLC chromatograms of DA_{inv}-product (left) and *f*-HM-SiR (right). λ_{ex} = 650 nm.

4.3 Synthesis of HM-SiR

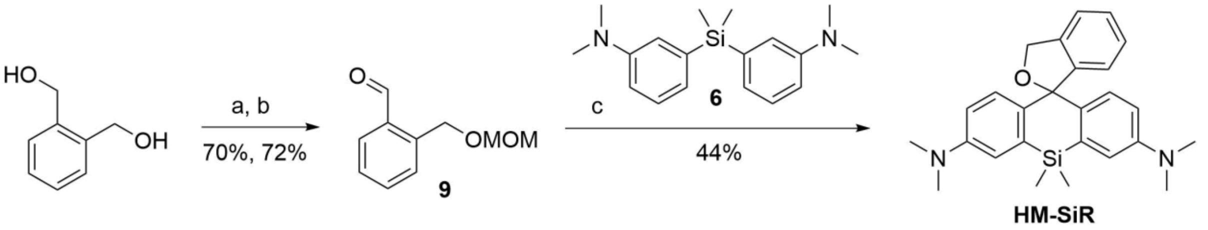

a: 1) NaH, THF, 2) MOMCl, b: DMP, DCM; c: 1) $AlBr_3$, 1,2-dichloroethane, 4 Å mol. sieve, 2) *p*-chloranil, 3) TFA, DCM

Scheme S2. Synthesis of HM-SiR via Friedel-Crafts reaction between **9** and diaryl silane nucleophile **6.**

(2-((Methoxymethoxy)methyl)phenyl)methanol (8)

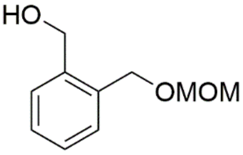

NaH (60% dispersion in mineral oil, 290 mg, 7.24 mmol) was suspended in dry THF (14 mL) under an argon atmosphere. At 0°C, 1,2-phenylendimethanol (1,0 g, 7.24 mmol) was added slowly. After complete addition, another 10 mL of dry THF were added. Subsequently, the reaction mixture was allowed to heat up to rt and stirred overnight. At the next day, chloromethyl methyl ether (0.55 mL, 7.24 mmol) was added slowly to the vigorously stirred suspension. After 1 h stirring, the mixture was diluted with water until formed NaCl was completely dissolved and extracted with EtOAc. The combined organic layers were washed with brine, dried over $MgSO_4$ and the solvent was removed *in vacuo*. Purification by flash column chromatography (silica, 0-25% EtOAc/cyclohexane) afforded the title product as a colorless oil (0.92 g, 70%).

^{1}H NMR (300 MHz, $CDCl_3$) δ 7.46 – 7.28 (m, 4H), 4.71 (s, 4H), 4.70 (s, 2H), 3.41 (s, 3H), 2.75 (t, J = 6.3 Hz, 1H). ^{13}C NMR (75 MHz, $CDCl_3$) δ 140.3, 135.7, 130.2, 129.6, 129.0, 128.2, 95.8, 68.0, 63.6, 55.8. HRMS (ESI^+) m/z 205.0835 calcd for $[C_{10}H_{14}NaO_3]^+$ ($M+Na^+$) 205.0835, 205.0836 found.

2-((Methoxymethoxy)methyl)benzaldehyde (9)

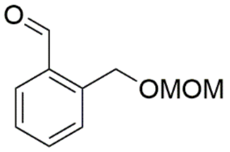

8 (240 mg, 1.32mmol) was dissolved in DCM (10 ml) and Dess-Martin periodinane (838 mg, 1,98 mmol) was added. The mixture was stirred for 0.5 h at rt until TLC monitoring indicated complete conversion. Subsequently, the solvent was evaporated and the mixture purified by flash chromatography (silica, 10% EtOAc/cyclohexane), yielding the title product (170 mg, 72%) as pink solid.

^{1}H NMR (300 MHz, $CDCl_3$) δ 10.24 (s, 1H), 7.90 – 7.85 (m, 1H), 7.69 – 7.57 (m, 2H), 7.53 – 7.45 (m, 1H), 5.03 (s, 2H), 4.77 (s, 2H), 3.42 (s, 3H). ^{13}C NMR (75 MHz, $CDCl_3$) δ 192.8, 140.6, 134.0, 133.7, 132.6, 128.6, 128.0, 96.4, 66.8, 55.7. HRMS (ESI$^+$) m/z 203.0679 calcd for $[C_{10}H_{12}NaO_3]^+$ (M+Na$^+$), 203.0684 found.

HM-SiR

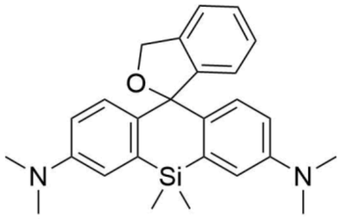

HM-SiR

3,3'-(Dimethylsilanediyl)bis(*N*,*N*-dimethylaniline) **6** (16.6 mg, 55.5 µmol) and **9** (20.0 mg, 111 µmol) were dissolved in anhydrous 1,2-dichloroethane (0.6 mL) in a Schlenk tube charged with activated molecular sieves (4 Å) under argon. $AlBr_3$ solution (1 M in dry dibromomethane, 83.2 µmol) was added dropwise and the mixture was stirred for 20 h at rt. Subsequently, *p*-chloranil (13.6 mg, 55.5 µmol) was added and after 2 h stirring, methanol (2 mL) was added and the mixture was filtered over a silica pad. The solvent was removed under reduced pressure and the blue solid residue was dissolved in dichloromethane (2 mL) at 0°C. After the dropwise addition of trifluoroacetic acid (180 µL, 2.34 mmol), the mixture was allowed to heat up to rt and stirred for 2h. The solvent was removed under reduced pressure and purification by flash chromatograph (silica, 1. DCM, 2. 20% ethyl acetate/DCM; 3. 1-8% MeOH/DCM with 2% acetic acid) afforded **HM-SiR** (13.4 mg, 44%) as a blue solid.

Note: Trifluoroacetic acid was titrated to NMR samples in order to shift the chemical equilibrium to the open hydroxyl derivative.
Prior to photophysical or cell experiments, HPLC purification was performed (20-90% Solvent B/Solvent A).

^{1}H NMR (500 MHz, CD_3CN) δ 7.68 (d, *J* = 7.4 Hz, 1H), 7.58 (t, *J* = 7.2 Hz, 1H), 7.45 (t, *J* = 7.5 Hz, 1H), 7.27 (d, *J* = 2.8 Hz, 2H), 7.13 (d, *J* = 7.9 Hz, 1H), 7.02 (d, *J* = 9.7 Hz, 2H), 6.68 (dd, *J* = 9.7, 2.8 Hz, 2H), 4.27 (s, 2H), 3.28 (s, 12H), 0.58 (s, 3H), 0.58 (s, 3H). ^{13}C NMR (126 MHz, CD_3CN) δ 155.2, 149.0, 142.1, 140.5, 138.1, 129.9, 129.9, 128.3, 128.2, 127.8, 122.0, 115.0, 111.0, 62.2, 41.3, -1.0, -1.2. HRMS (ESI$^+$) m/z 415.2200 calcd for $[C_{26}H_{31}N_2OSi]^+$ (M+H$^+$), 415.2209 found.

4.4 Other Compounds

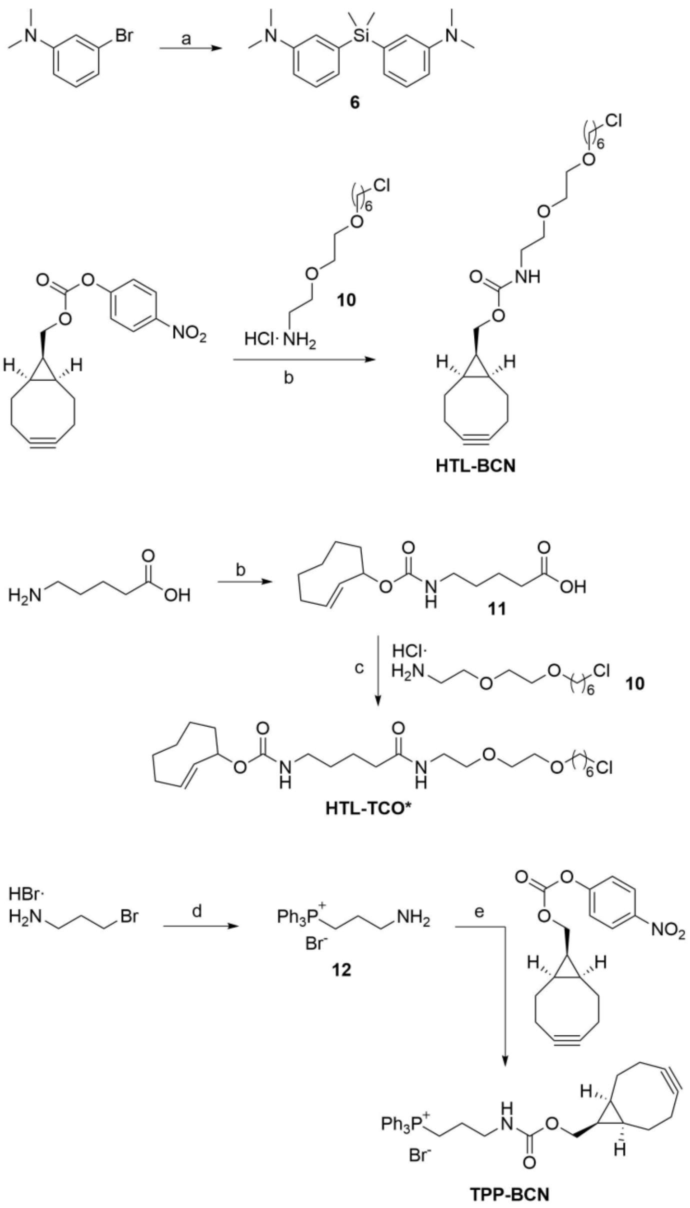

a: 1) *s*-BuLi, 2) Me_2SiCl_2, THF; b: DIPEA, DMF; c: DIPEA, HCTU, DMF; d: PPh_3, MeCN; e: DIPEA, DMF.

Scheme S3.

6,[7] **HTL-BCN**,[8] **10**,[7] **11**[7] and **12**[9] were synthesized according to literature procedures.

HTL-TCO*

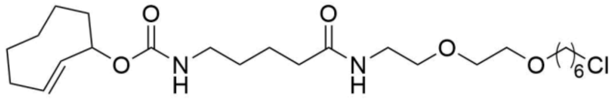

11 (12 mg, 45 µmol), DIPEA (8 µL, 135 µmol) and HCTU (20 mg, 47 µmol) were dissolved in dry DMF (1 mL) at rt and stirred for 15 min. Subsequently, a solution of **10** (10 mg, 47 µmol) in dry DMF (1 mL) was added and the mixture was stirred for 3 h. The solvent was concentrated *in vacuo* and the crude product was purified by HPLC (10%-70% solvent B/Solvent A in 50 min) yielding the title product (18 mg, 85%).

^{1}H NMR (300 MHz, Methanol-d_4) δ 5.91 – 5.76 (m, 1H), 5.55 (dd, J = 16.5, 1.9 Hz, 1H), 5.23 (s, 1H), 3.63 – 3.45 (m, 10H), 3.35 (t, J = 5.5 Hz, 2H), 3.11 (t, J = 6.7 Hz, 2H), 2.51 – 2.39 (m, 1H), 2.22 (t, J = 7.3 Hz, 2H), 2.11 – 1.27 (m, 21H), 1.23 – 1.08 (m, 1H), 0.94 – 0.80 (m, 1H). ^{13}C NMR (75 MHz, CD_3OD) δ 176.0, 158.4, 133.0, 132.5, 75.0, 72.2, 71.2, 71.2, 70.6, 45.7, 41.7, 41.2, 40.4, 37.1, 36.8, 36.6, 33.8, 30.5, 30.4, 30.1, 27.7, 26.5, 25.2, 24.2. HRMS (ESI$^+$) m/z 497.2753 calcd for [$C_{24}H_{43}ClN_2NaO_5^+$] (M+Na$^+$), 497.2774 found.

TPP-BCN

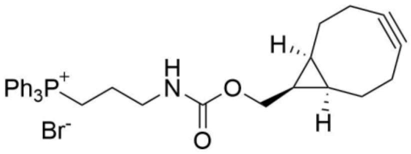

DIPEA (9.9 µL, 57 µmol) was added to a mixture of ((1*R*,8*S*,9*s*)-bicyclo[6.1.0]non-4-yn-9-yl)methyl (4-nitrophenyl) carbonate (6.00 mg, 19.0 µmol) and **12** (11.4 mg, 28.5 µmol) in anhydrous dimethylformamide (0.4 mL) at 0°C and stirred for 1h. Subsequently, the ice bath was removed and after 2h stirring at rt, water (2 mL) and dichloromethane (4 mL) was added. The aqueous phase was extracted with dichloromethane (3 x 2 mL) and combined organic layers were dried over Na_2SO_4. Volatiles were removed under reduced pressure and an analytical sample was purified by HPLC (20-90% Solvent B/Solvent A).

^{1}H NMR (500 MHz, CD_3OD) δ 7.92 – 7.88 (m, 3H), 7.82 – 7.74 (m, 12H), 3.95 (d, J = 7.0 Hz, 2H), 3.43 – 3.35 (m, 2H), 3.27 (t, J = 6.5 Hz, 2H), 2.37 – 2.29 (m, 2H), 2.26 – 2.16 (m, 2H), 2.12 – 2.05 (m, 2H), 1.90 – 1.81 (m, 2H), 1.41 – 1.28 (m, 2H), 0.76 – 0.69 (m, 2H), 0.64 (dt, J = 12.0, 6.5 Hz, 1H). HRMS (ESI$^+$) m/z 496.2400 calcd for [$C_{32}H_{35}NO_2P^+$] (M$^+$), 496.2411 found.

Miscellaneous SiR fluorophores

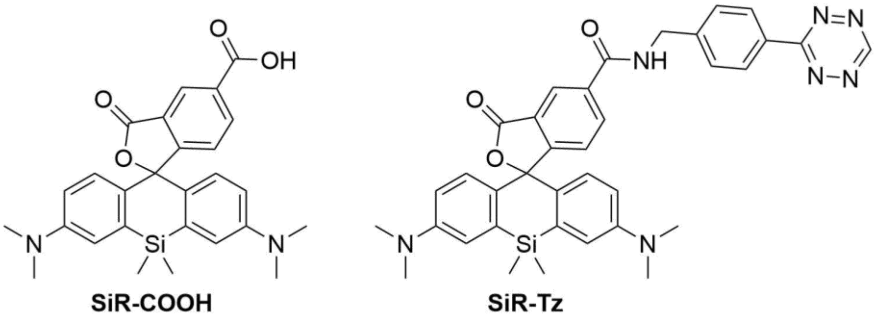

Figure S6

SiR-COOH and **SiR-Tz** are commercially available and can be purchased from Spirochrome Ltd., Stein am Rhein, Switzerland (cat. numbers: SC004 and SC006).

5 In Vitro Protein Labeling

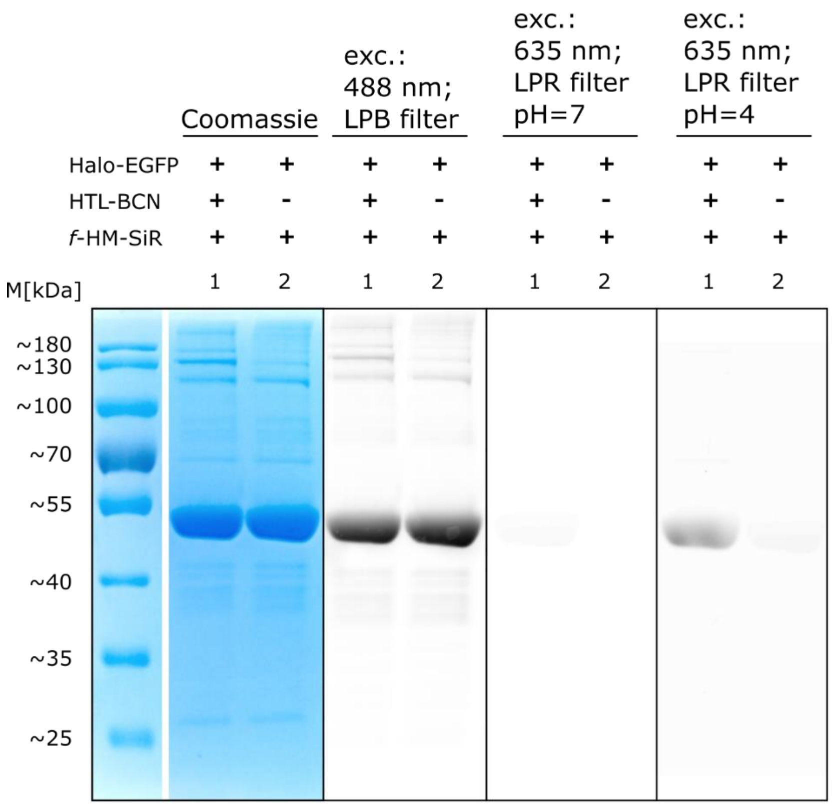

Figure S7. In vitro labeling of EGFP-BCN using *f*-HM-SiR. In gel fluorescence shows selective labeling of EGFP and confirms the pH-sensitivity of the protein-bound dye.

EGFP-HaloTag (5 µM) was treated with HTL-BCN (25 µM, from a 2.5 mM DMSO stock solution) in PBS (100 µL, pH 7.4) and incubated at 37 °C for 1 h. The BCN-functionalized protein solution was filled up to 500 µL with PBS (pH 7.4) and transferred into Amicon Ultra-0.5 Centrifugal Filter Units with 10 kDa cutoff (Merck KGaA) and centrifuged according the manufacturers' protocol. The flowthrough was discarded. The cartridge was filled up to 500 µL with PBS (pH 7.4), centrifuged again and the flowthrough was discarded. This step was repeated once. The retentate was recovered and the protein concentration was calculated from the absorbance of the concentrated protein solution at 280 nm.

The washed protein (5 µM) was then labeled with *f*-HM-SiR (25 µM, from a 1 mM DMSO stock solution) in PBS (20 µL, pH 7.4) at 37 °C for 1 h. For a negative control, DMSO was added instead of *f*-HM-SiR. To quench the reaction after 1 h (E)-Cyclooct-4-en-1-ol (axial-TCO) (2.5 mM) was added and incubated at RT for 5 min. The samples were treated with reducing SDS-sample buffer at 37 °C for 5 min and loaded onto a SDS gel with 10 % (w/v) acrylamide concentration. The gel was run at a constant voltage of 150 V for 80 min, rinsed briefly with water and treated with PBS (pH 7.0) for 30 min. The gel was mounted between

translucent foils and scanned with a Typhoon FLA 9500 fluorescence scanner (GE Healthcare Life Sciences) with 635 nm laser excitation and ≥ 665 nm fluorescence detection (LPR filter) or 488 nm laser excitation and ≥ 515 nm fluorescence detection (LPB filter). The gel was then treated with PBS (pH 4.0) for 30 min and scanned with the fluorescence scanner again. The gel was stained with colloidal Coomassie Brilliant Blue[10], destained with an aqueous AcOH solution (10 %, v/v) and scanned using a document scanner.

6 Cell Culture

HeLa and COS-7 cells were cultured in full growth medium based on Phenol Red-free Dulbecco's modified eagle medium (DMEM) supplemented with 10 % fetal calf serum (FCS), 2 mM L-Glutamine and 1 mM sodium pyruvate (all Gibco/Life Technologies) in humidified atmosphere at 37 °C and 5 % CO_2. Cells were routinely sub-cultured upon reaching 90 % confluency.

7 Preparation of Cells for Imaging

Cells were prepared for imaging by seeding $7.5 \cdot 10^3$ cells*cm^{-2} into #1 LabTek chambered coverslides (ThermoFisher Scientific) 48 hours prior to imaging. LabTek coverslides were cleaned with 0.1 M hydrofluoric acid before seeding of cells. The cloning of expression plasmid H2A-HaloTag and TOMM20-HaloTag and TOMM20-mCherry-HaloTag is described in Hauke *et al*.[11] Transient transfection of COS-7 cells was performed using FuGENE® HD transfection reagent (Promega) 24 hours prior to imaging following transfection guidelines provided by the manufacturer. HeLa cells were transfected 20-24 hours prior to imaging using Lipofectamine® 2000 (ThermoFisher Scientific) or FuGene® HD transfection reagent. For transfection of a single LabTek well using Lipofectamine® 2000, 0.4 µg plasmid DNA were diluted in 50 µl serum-free OPTI-MEM (Gibco/ Life Technologies) followed by addition of 0.6 µl Lipofectamine® 2000. After incubation for 20 minutes, the transfection mix was added to the corresponding LabTek well. Transient transfection with FuGene® HD was performed by diluting 0.2 µg plasmid DNA in 10 µl serum-free OPTI-MEM followed by addition of 0.6 µl FuGene® HD. After incubation for 5 minutes, the transfection mix was added to the LabTek.

Labeling of mitochondria using TPP-BCN

For imaging of mitochondria using TPP-BCN, cells were loaded with 10 µM TPP-BCN diluted in serum-free growth medium at 37 °C for 30 minutes followed by two 15 minute washing steps in full growth medium, all at 37 °C. Cells were then labeled with 2 µM ***f*-HM-SiR** for 30 minutes in serum-free growth medium. Prior to imaging medium containing f-HM-SiR was replaced with full growth medium.

Labeling of HaloTag fusion proteins

Cells expressing HaloTag fusion proteins were labeled with ***f*-HM-SiR** by first loading cells with HTL-TCO* or HTL-BCN at 10 µM for 30 minutes in serum-free growth medium. Unbound HTL-TCO* or HTL-BCN was removed by washing twice with full growth medium for 15 minutes each followed by a single 30 minute washing step with full growth medium. If indicated, HaloTag fusion protein expressing cell lines were counter stained with HTL-TMR to serve as co-localization and transfection control. For this, 15 nM HTL-TMR (Promega) were added to the corresponding samples during the first 15 minute washing step after incubation with HTL-TCO* or HTL-BCN.

Chemical fixation was performed with freshly prepared aqueous formaldehyde solution (3.7 % w/v PFA in PBS).

8 Fluorescence Microscopy

All imaging was performed on a Nikon Ti-E inverted microscope equipped with TIRF illumination optics, a 100x 1.49 NA Apo-TIRF objective lens and perfect focus (PFS) autofocus system (all Nikon). A fiber-coupled multi-laser engine equipped with 405, 488, 561 and 640 nm lasers (TOPTICA Photonics) was used for excitation. The excitation lasers were coupled into the objective off the central axis of the objective to achieve HiLO illumination.[11] Emitted fluorescence was separated from excitation light using a 405/488/561/640 nm quad-band dichroic mirror and further filtered with 605/70 nm or 685/70 nm bandpass filters for TMR and ***f*-HM-SiR** signal respectively (all AHF Analysetechnik). An emCCD camera (iXon Ultra 897, Andor Technology) was used for data acquisition. Typically, the central 256x256 pixel area was read out with an exposure time of 20 ms and a final pixel size of 150 nm/px within the sample. Microscopy hardware was controlled with open source software package Micro-Manager.[12] Imaging was performed at 21 °C in full growth medium as previously described.[13]

The illumination power density was determined by first measuring the transmitted power after the objective using a handheld power meter (Thorlabs) at illumination conditions identical to those used for imaging and then normalized to the illuminated area. The illuminated area was determined by recording images from homogenous samples and fitting a two-dimensional Gaussian function to the acquired images. Unless stated otherwise, ***f*-HM-SiR** was excited at a peak power density of 1.2 kW/cm^{-2}.

9 Supporting Microscopy Figures

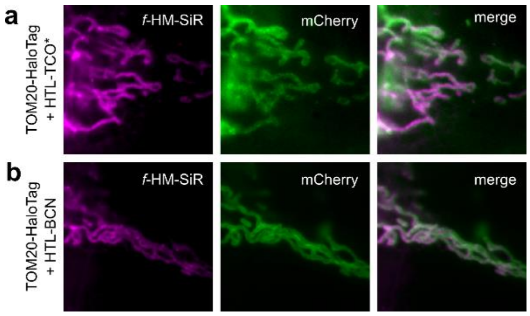

Figure S8. COS-7 cells transiently expressing TOM20-mCherry-HaloTag were incubated with different HTL-dienophile ligands and subsequently labeled with *f*-HM-SiR. HTL-TCO* (a) or HTL-BCN (b) were introduced as dienophile at a concentration of 2 µM. *f*-HM-SiR was visualized by averaging 100 raw images.

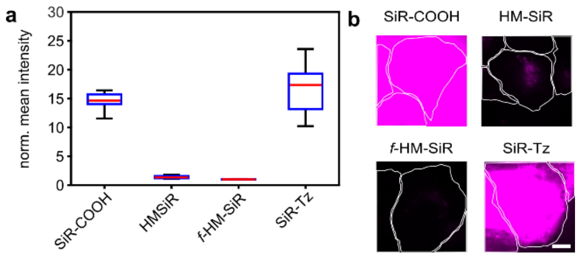

Figure S9. Background intensity of otherwise untreated HeLa cells incubated with various SiR derivatives (2 µM) under no-wash imaging conditions. **a)** Intensities were measured within segmented cells (see methods). 20 cells per condition. **b)** Representative images for conditions shown in a). White lines represent cell boundaries. Scale bar in b) 10 µm.

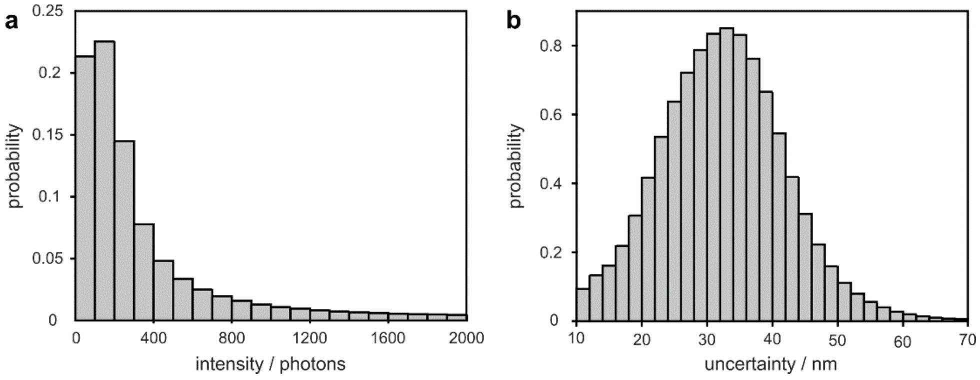

Figure S10. Photon counts per localization (median 235.4 photons) and corresponding localization uncertainty (median 32.4 nm) for individual *f*-HM-SiR blink events in HeLa cells loaded with TPP-BCN (10 µM) as shown in Fig. 4d-h. For raw data from the same dataset see movie S4.

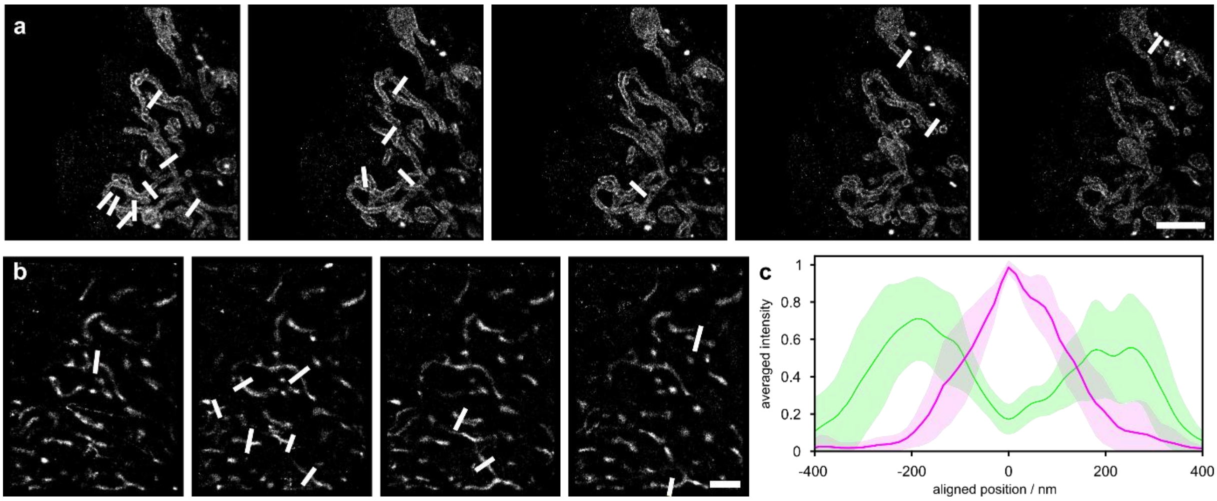

Figure S11. Line profiles used for characterization of cross-sectional profiles of mitochondria imaged with *f*-HM-SiR. **a)** Line profiles for labeling with TOM20-HaloTag and HTL-BCN as shown in fig. 3a. Line profiles were recorded in reconstructions at different time points. **b)** Line profiles for labeling with TPP-BCN as shown in fig. 4d. Line profiles were recorded in reconstructions at different time points. **c)** Overlayed averaged cross-sectional profiles for labeling of the outer mitochondrial membrane with TOM20-HaloTag (green) and the mitochondrial matrix with TPP-BCN (magenta) as shown in figs. 3e and 4e. Scale bars: a) 5 µm, b) 2 µm.

10 SMLM Reconstructions

All raw data used for SMLM analysis was pre-processed using a three level Haar wavelet kernel (HAWK) transform as implemented by Marsh et al.[14] to separate spatially overlapping single-molecule signals. Single-molecule localization in the transformed raw data was then performed using the ImageJ plugin thunderSTORM[15] with input parameters adjusted to the imaging setup used. Briefly, images were wavelet filtered, thresholded using the local maximum approach and fitted with sub-pixel accuracy by Maximum likelihood estimation without multi-emitter fitting as implemented in thunderSTORM. Reconstructions of the obtained localizations were computed by filtering localizations based on width and amplitude of the Gaussian fit and plotting each localization as individual Gaussian with a width corresponding to the localization uncertainty for this localization into an image with a pixel size of 15 nm. Dynamic structures were visualized by additionally filtering localizations used for individual reconstructions based on their time of appearance. For sliding window reconstructions (movies S1, S2, S3) overlapping time windows were used for computing individual reconstructions. An additional weighted sum temporal color coding of localization appearance within a single reconstruction (Fig. 4h, movie S3) was applied using the ImageJ plugin "Temporal Color Code" originally authored by Kota Miura[1] and extended by Eugene Katrukha[2].

11 Localization Precision Measurements

The localization uncertainty for ***f*-HM-SiR** under the applied imaging conditions (see section above) was determined using thunderSTORM and raw data without HAWK transformation. Deliberately under-labeled samples were imaged under otherwise identical preparation and imaging conditions in live cells directly after calibration of the electron-multiplying gain register of the emCCD camera used.

12 Evaluation of Acute Phototoxicity

Acute phototoxicity was assayed by inspecting mitochondrial dynamics in the presence or absence of 640 nm illumination at 1.2 kW/cm^{-2} as it was typically employed for imaging ***f*-HM-SiR**. For this, HeLa cells were transiently transfected with a plasmid expressing

---

[1] https://github.com/fiji/fiji/blob/master/plugins/Scripts/Image/Hyperstacks/Temporal-Color_Code.ijm

[2] Footnote:https://gist.github.com/ekatrukha/4f991ba68e50deac38da7561d8a0b085

TOMM20-mCherry-HaloTag and imaged under low-intensity 561 nm illumination before and after 640 nm illumination. No changes in mitochondrial dynamics were observed if 640 nm illumination was applied (movie S5).

13 Image Processing and Statistical Analysis

All images were processed and prepared for presentation using FIJI.[16] Diffraction-limited images from self-blinking ***f*-HM-SiR** were obtained by averaging acquired image stacks consisting of at least 100 frames. All images were adjusted for brightness and contrast. Unless stated otherwise, images for direct comparison between conditions were adjusted to identical brightness and contrast settings. Peak-to-peak distances for TOM20-conjugated ***f*-HM-SiR** were computed by fitting line profiles across individual mitochondria with a double-Gaussian function using MATLAB and determining the distance between the centers of both Gaussians. The width of mitochondria imaged using TPP-BCN conjugated ***f*-HM-SiR** was determined by fitting line profiles across individual mitochondria with a Gaussian function. Sigma obtained from these fits was then used to compute the full width at half maximum (FWHM). Averaged line profiles for TOM20- and TPP-based imaging were generated by aligning all quantified profiles to the central minimum of the double-Gaussian fit or the maximum of the single-Gaussian fit respectively. Box plots were generated from extracted data using MATLAB. The red line denotes the median of the population and the boxed region includes all data points between the first (Q1) and third quartile (Q3). Whiskers denote 1.5x the interquartile distance (Q3-Q1).

14 Evaluation of Non-Specific Labeling

Unspecific background labeling (Fig. 2d,e and Fig. S9) was quantified by incubating HeLa cells with indicated SiR derivatives at a concentration of 2 µM for 30 minutes. To facilitate automated segmentation of cell bodies in images, cells were counter-stained with 20 nM MitoTracker™ Green FM (ThermoFisher Scientific).

Background localization rates using fluorogenic ***f*-HM-SiR** and non-fluorogenic **HM-SiR** (Fig. 3h) were determined by labeling untreated HeLa cells as described above for imaging with TPP-BCN. Images were acquired with identical acquisition settings used for imaging specifically bound ***f*-HM-SiR**.

15 NMR Spectra

^{1}H NMR of compound **1** (CD_3CN)

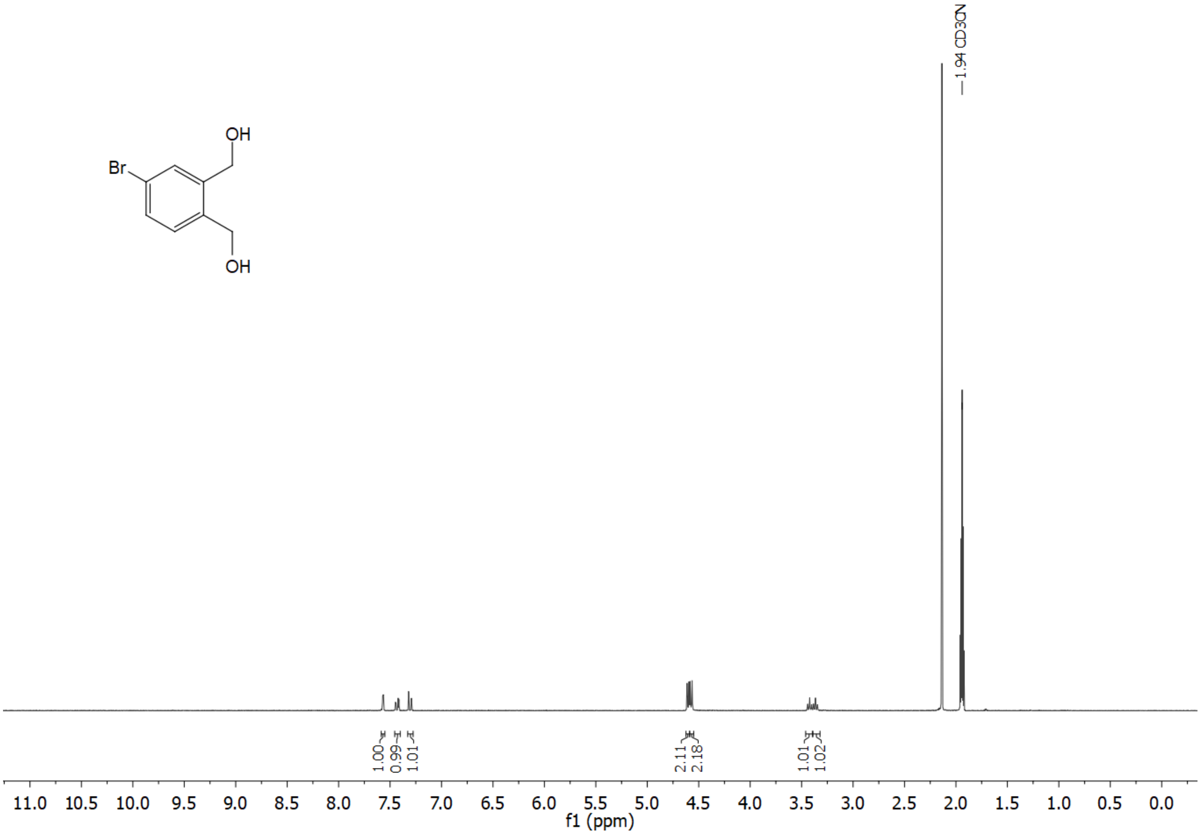

^{1}H NMR of compound **2a** ($CDCl_3$)

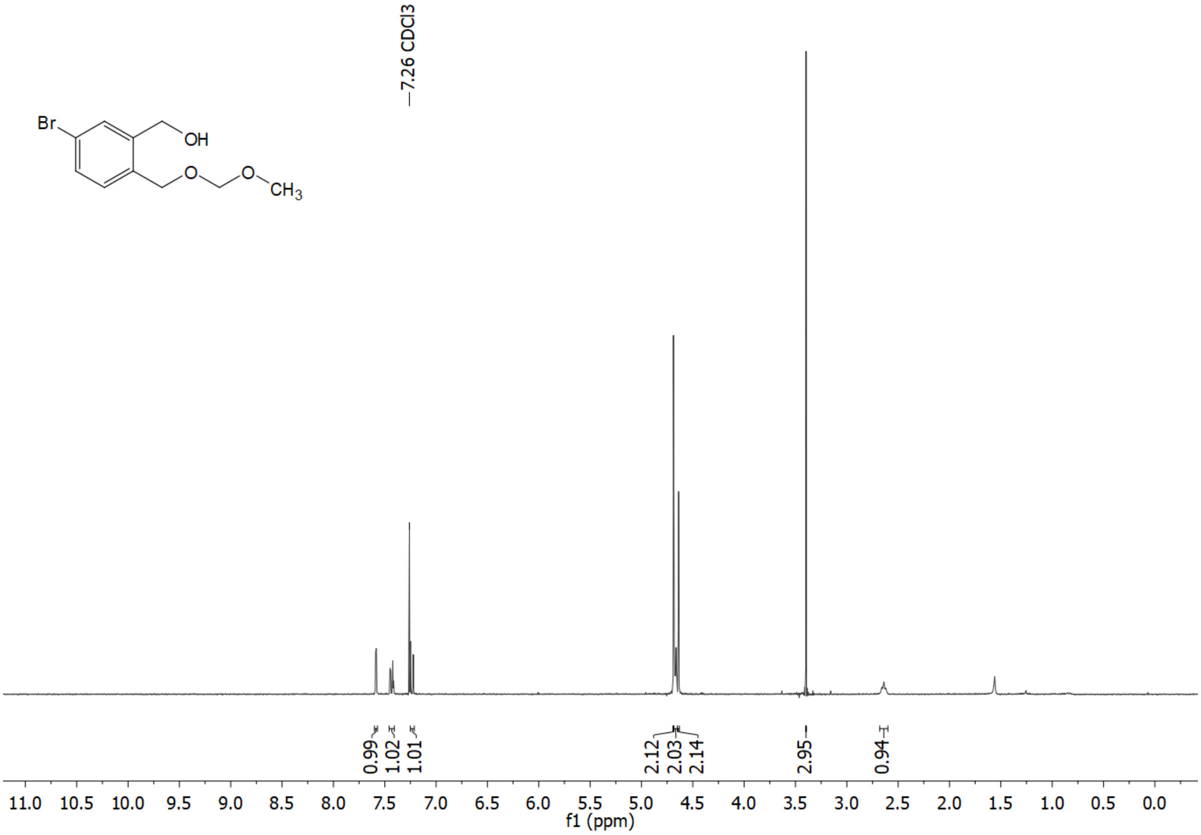

^{13}C NMR of compound **2a** ($CDCl_3$)

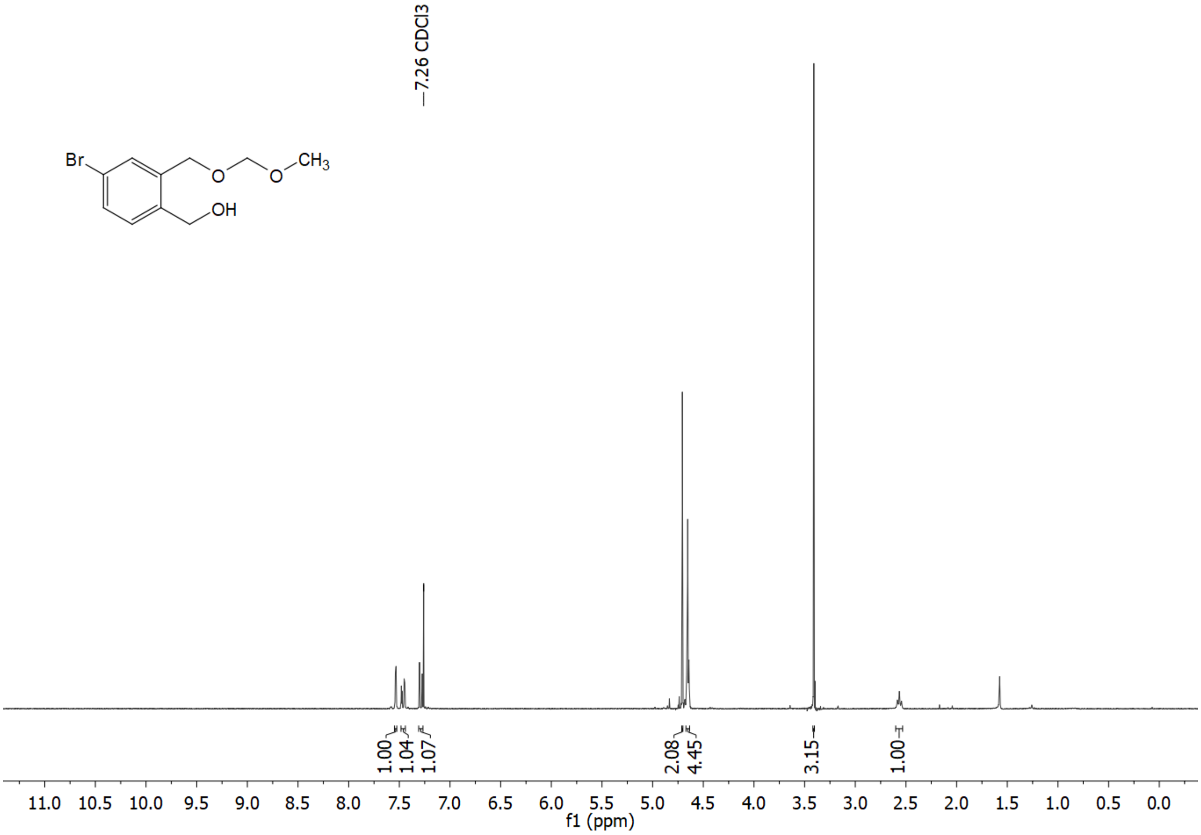

^{1}H NMR of compound **3** ($CDCl_3$)

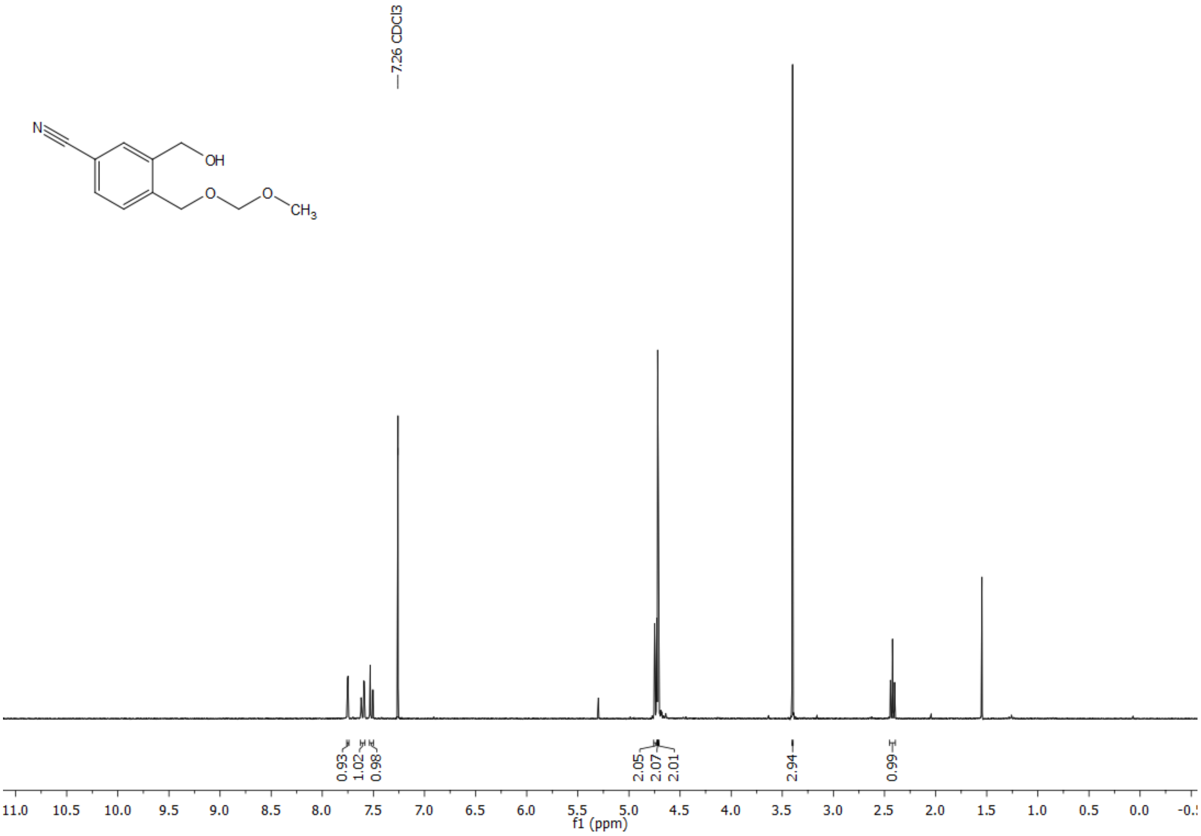

^{13}C NMR of compound **3** ($CDCl_3$)

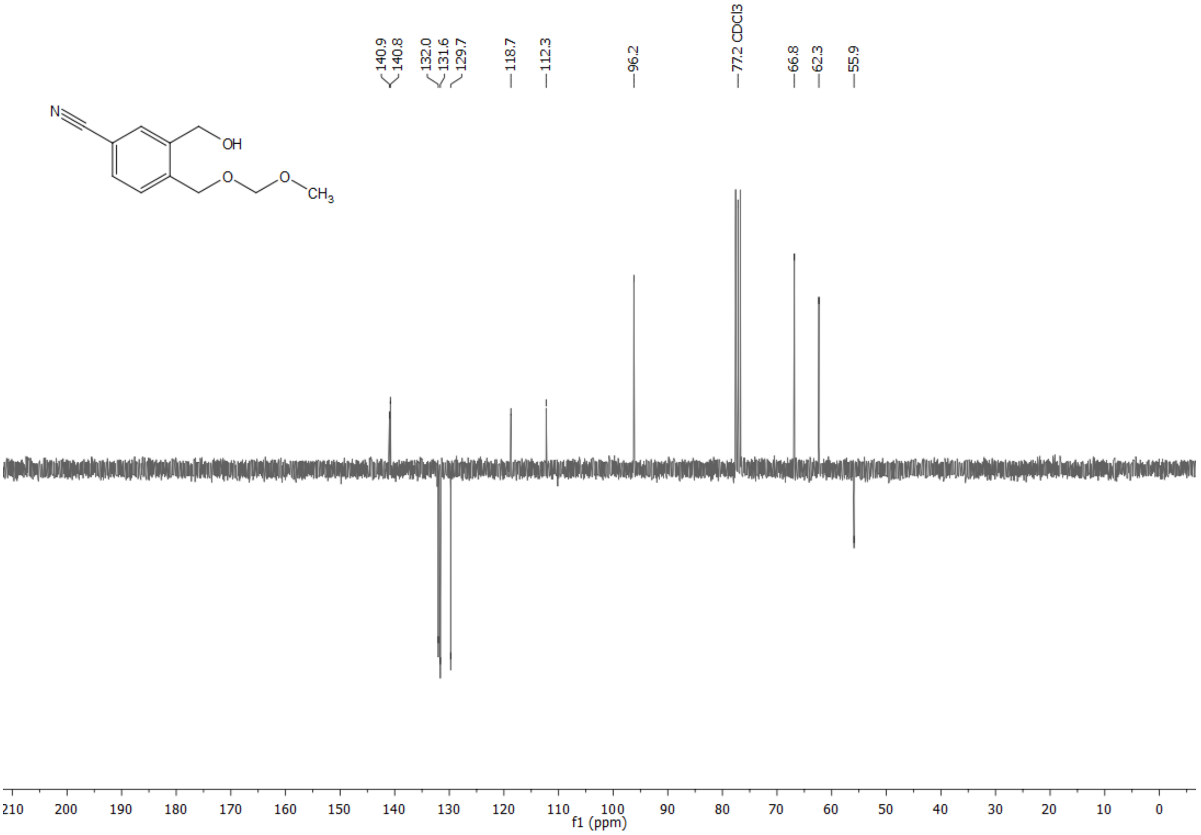

^{1}H NMR of compound **4** ($CDCl_3$)

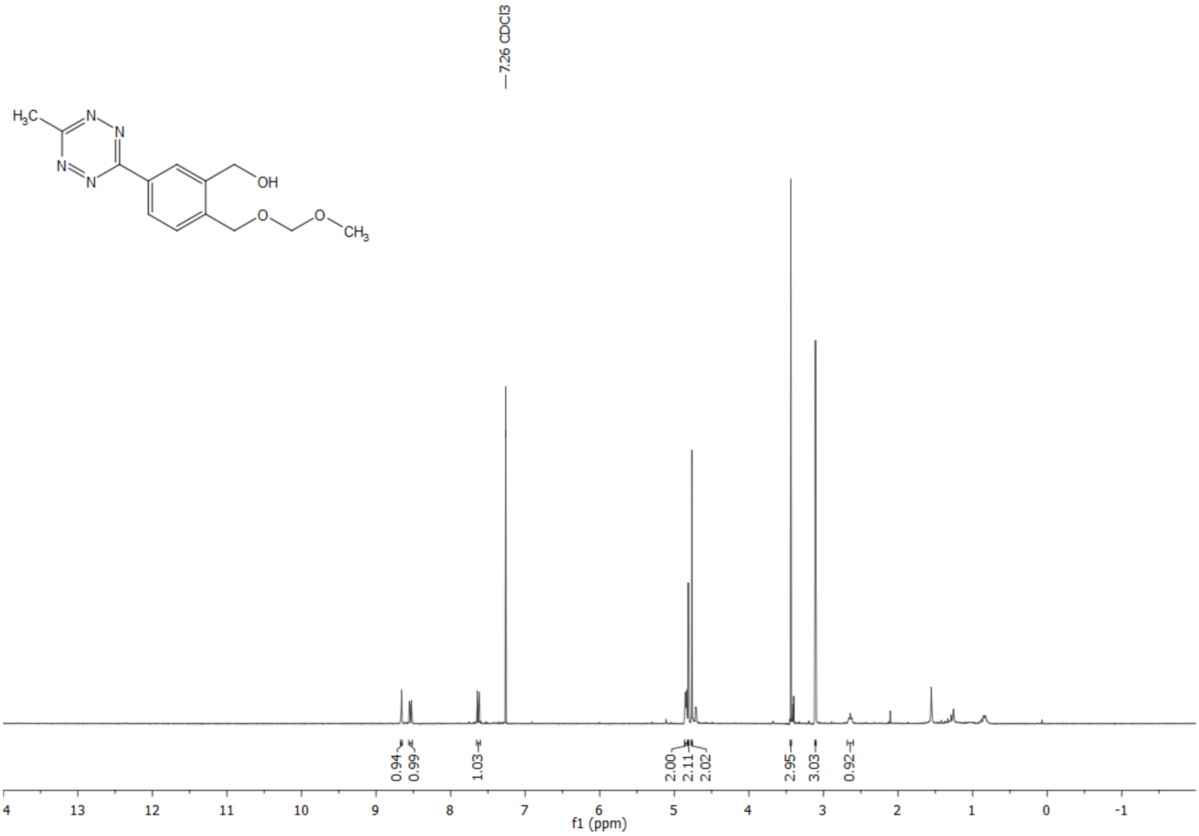

^{13}C NMR of compound **4** ($CDCl_3$)

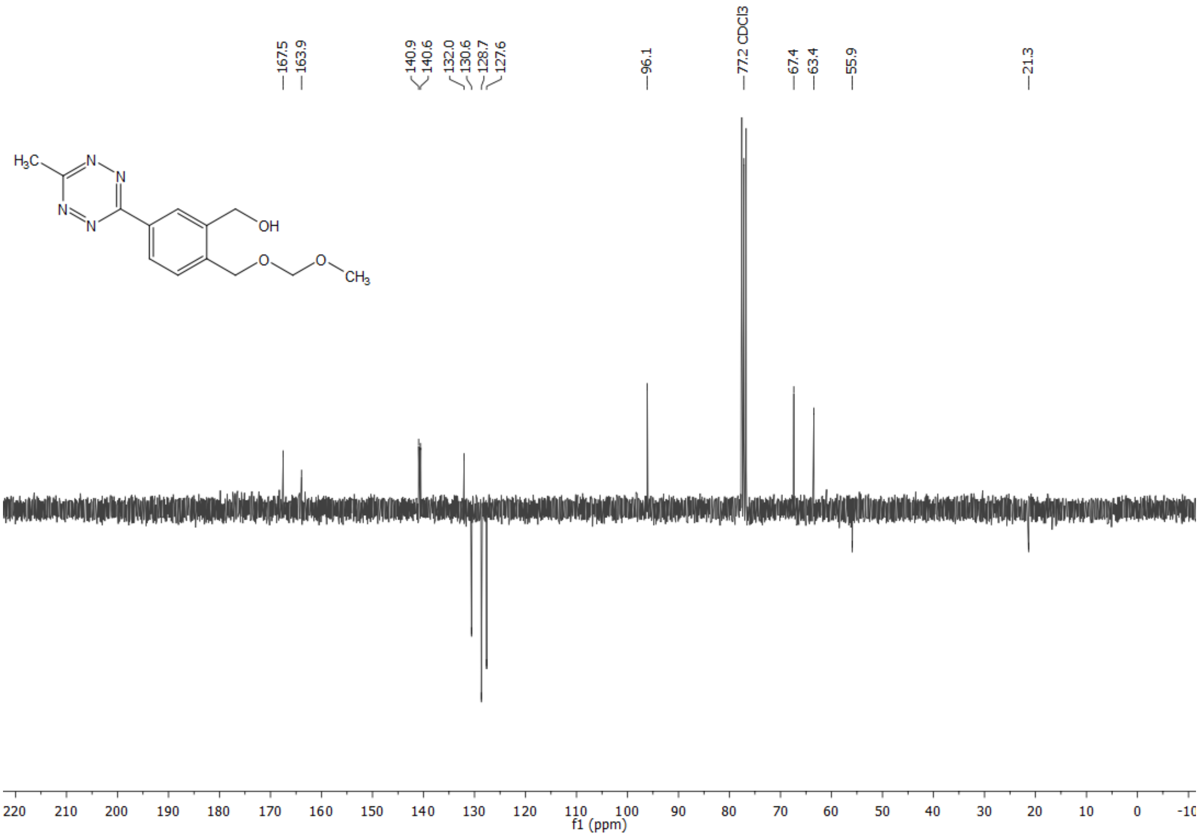

^{1}H NMR of compound **5** ($CDCl_3$)

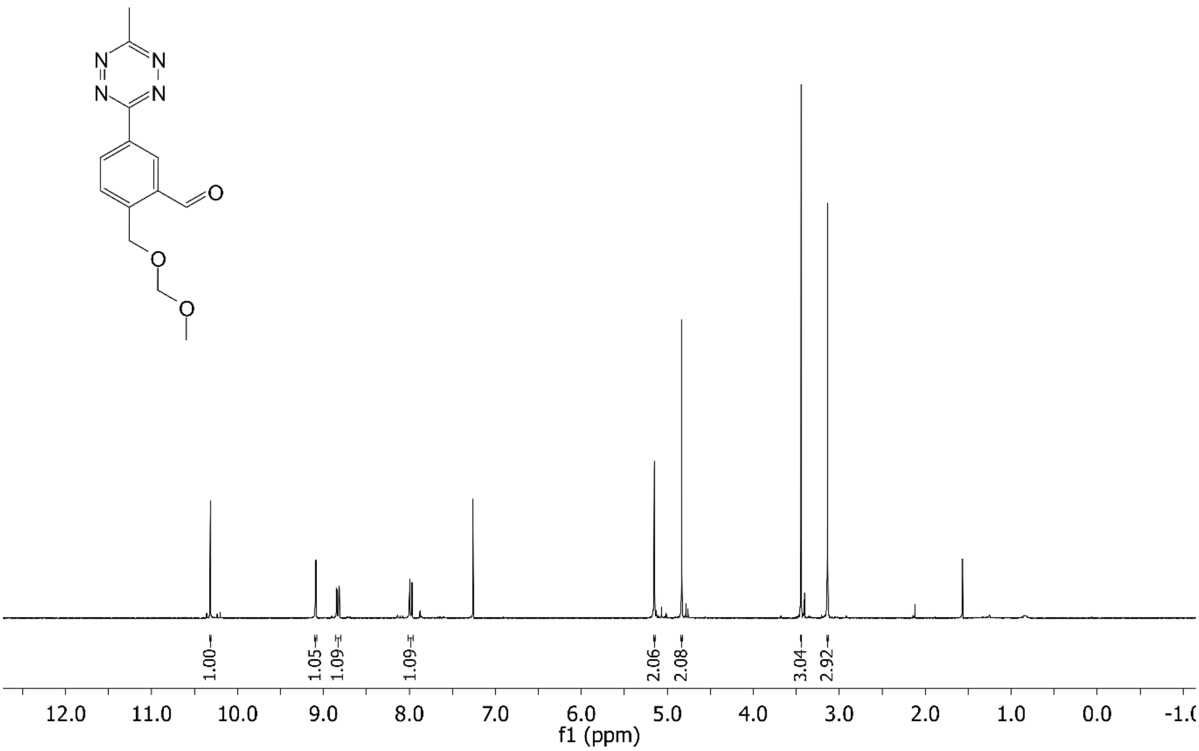

^{13}C NMR of compound **5** ($CDCl_3$)

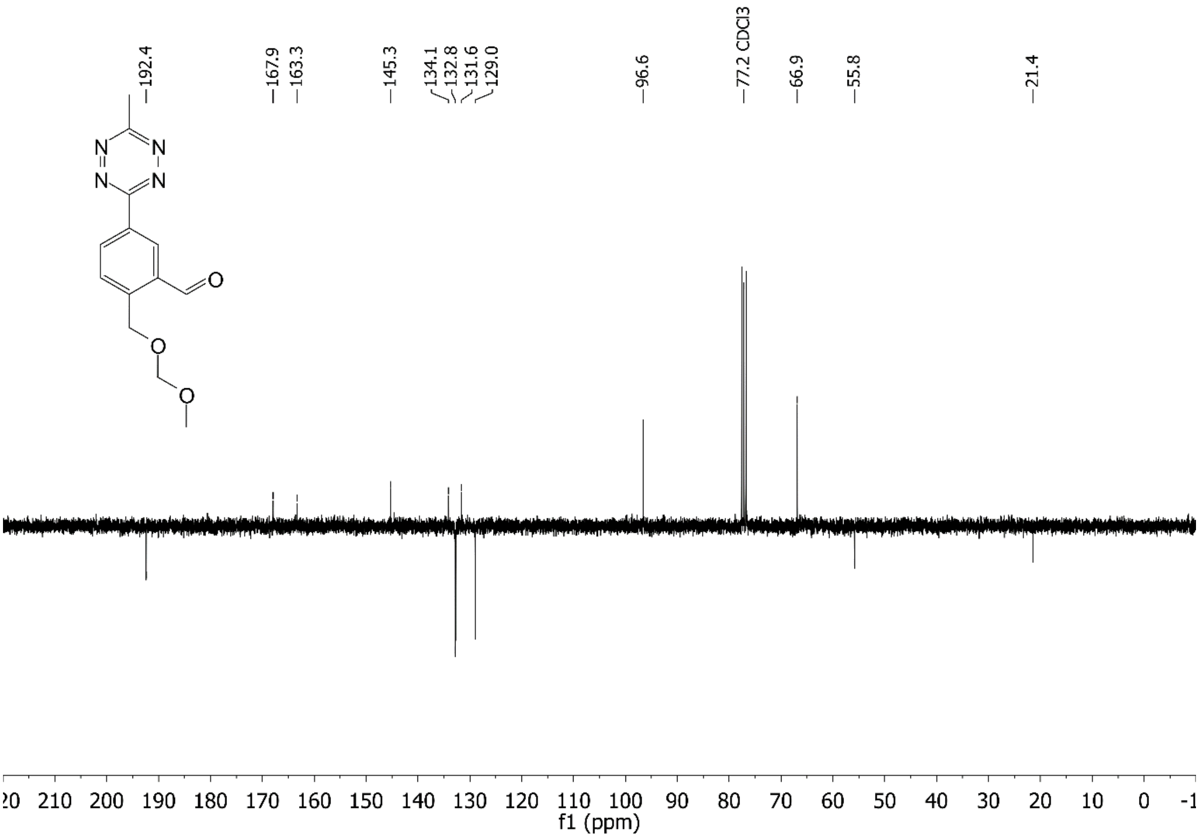

^{1}H NMR of **7** (CD_3CN)

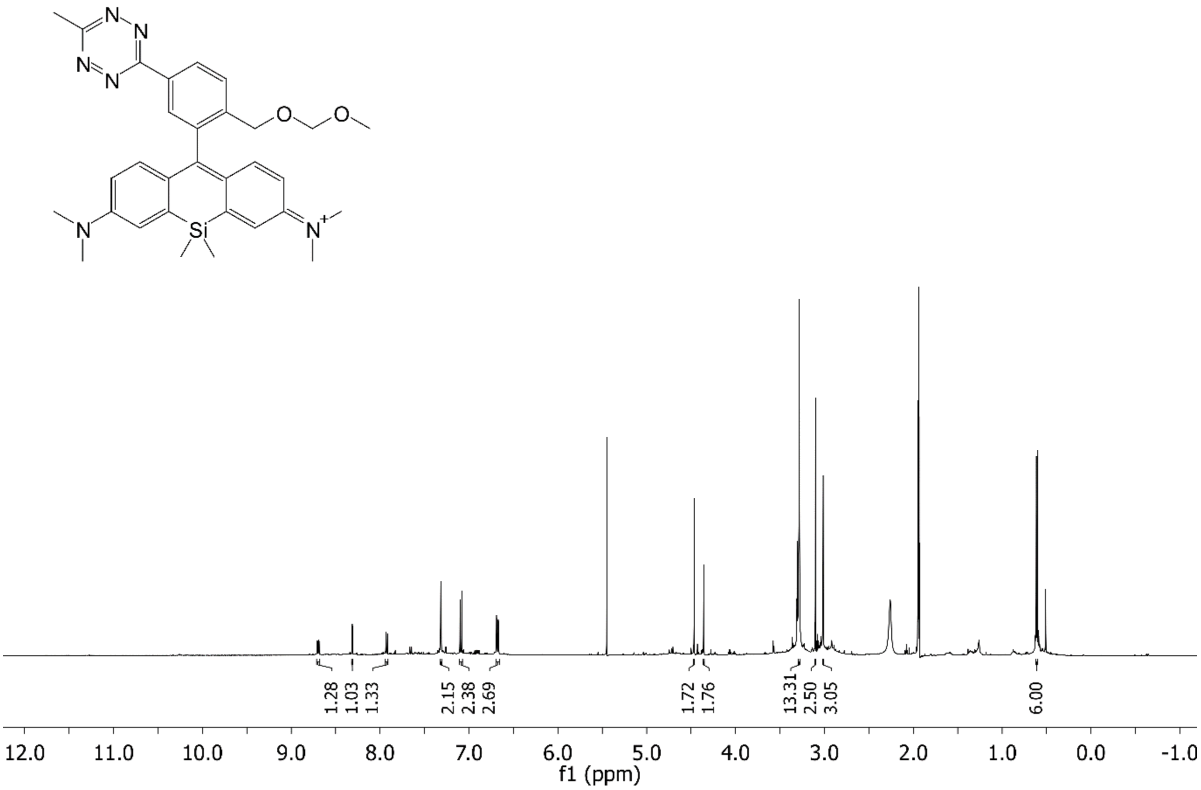

^{13}C NMR of **7** (CD_3CN)

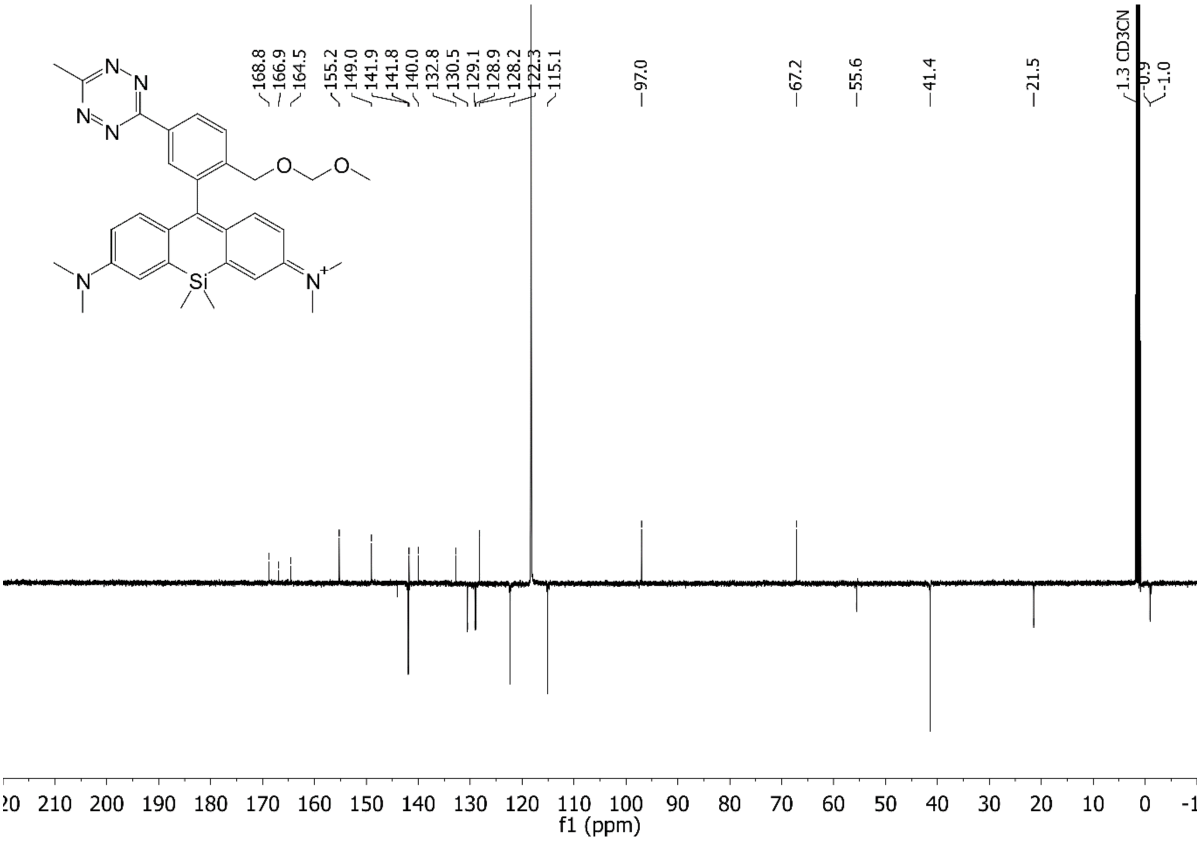

^{1}H NMR of ***f*-HM-SiR** (CD_3CN)

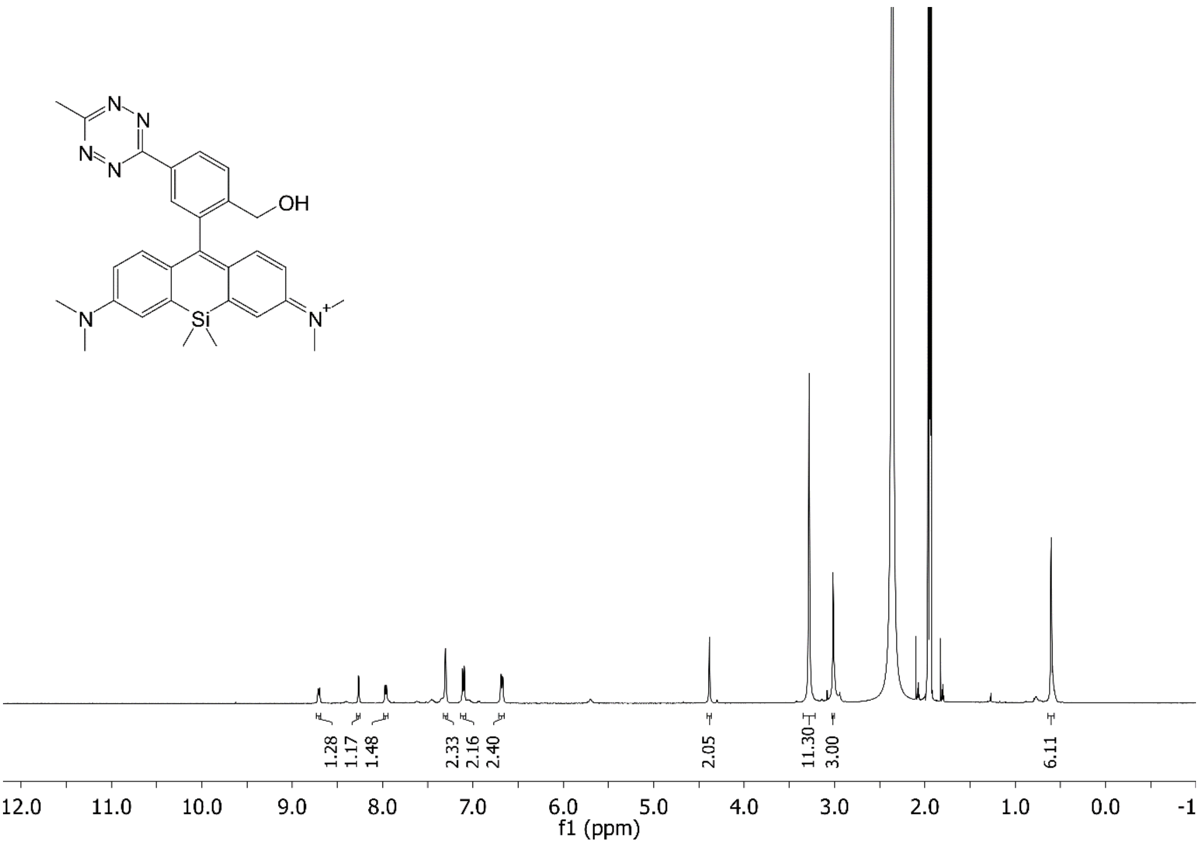

^{13}C NMR of compound ***f*-HM-SiR** (CD_3CN)

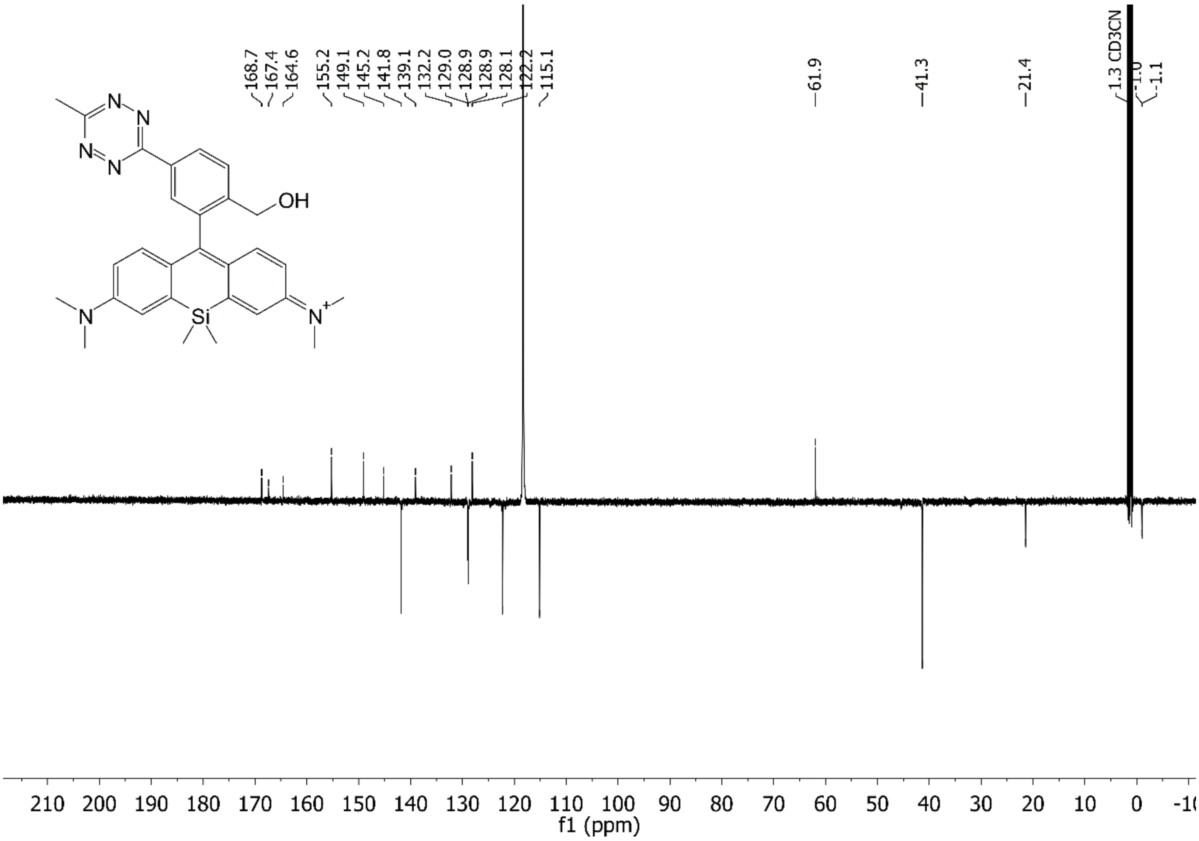

^{1}H NMR of **8** ($CDCl_3$)

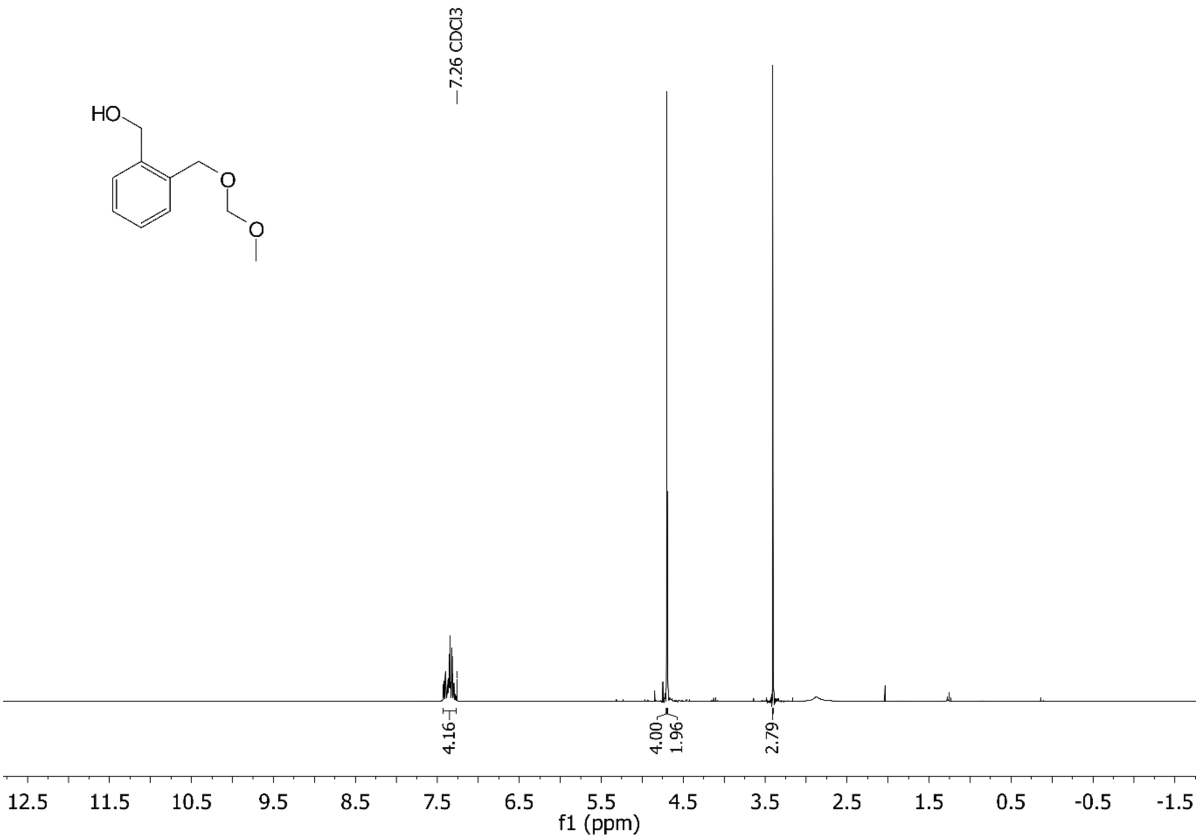

^{13}C NMR of of **8** ($CDCl_3$)

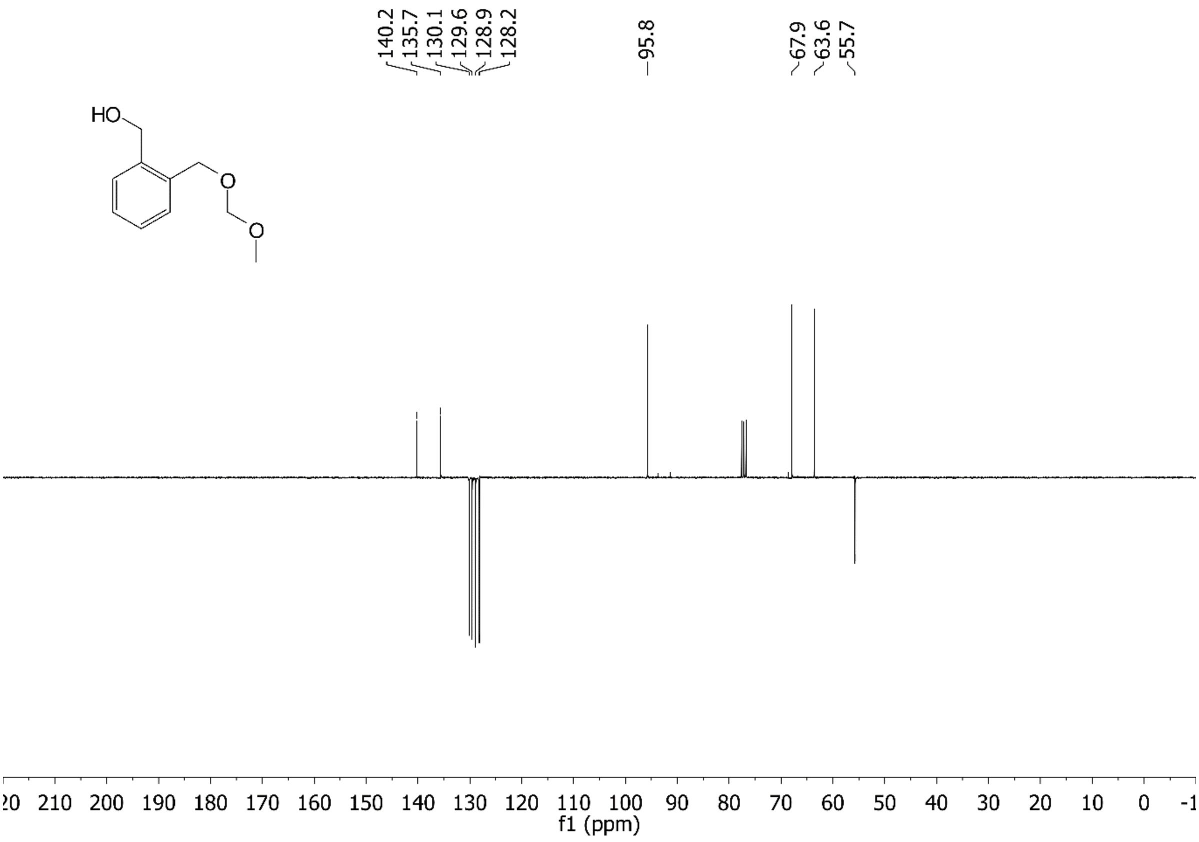

^{1}H NMR of **9** ($CDCl_3$)

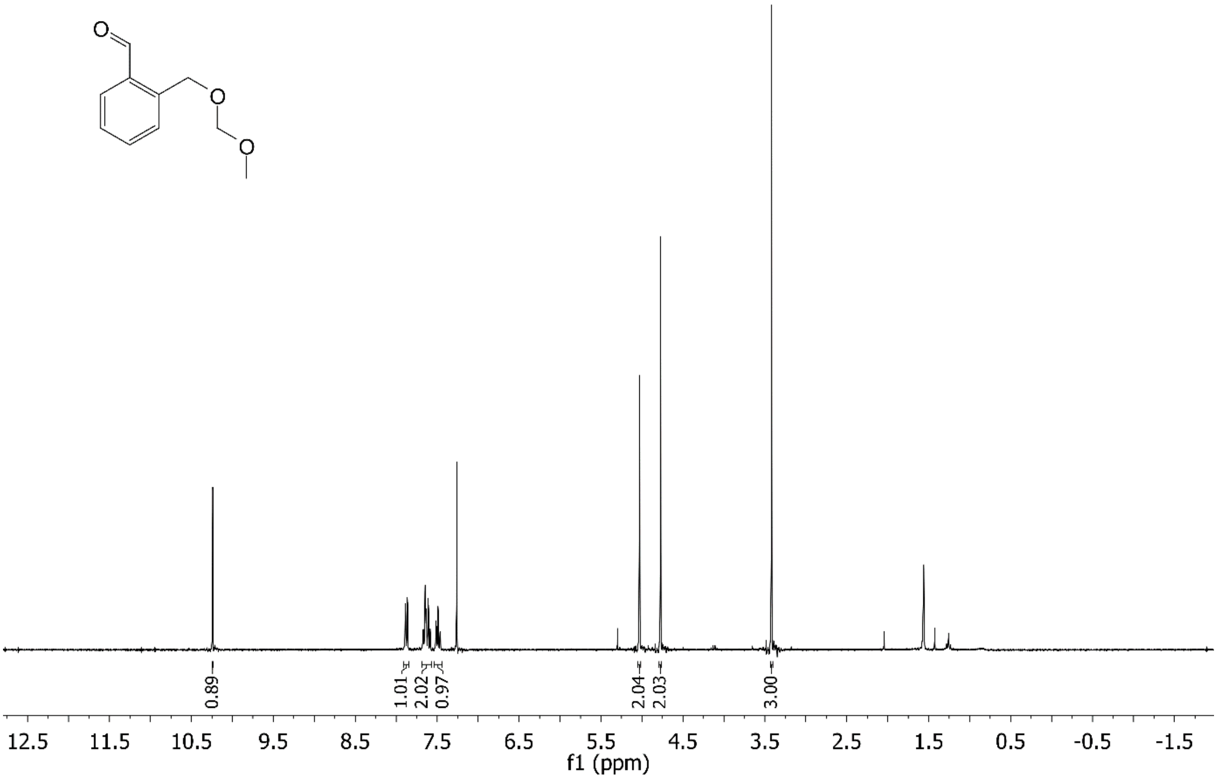

^{13}C NMR of of **9** ($CDCl_3$)

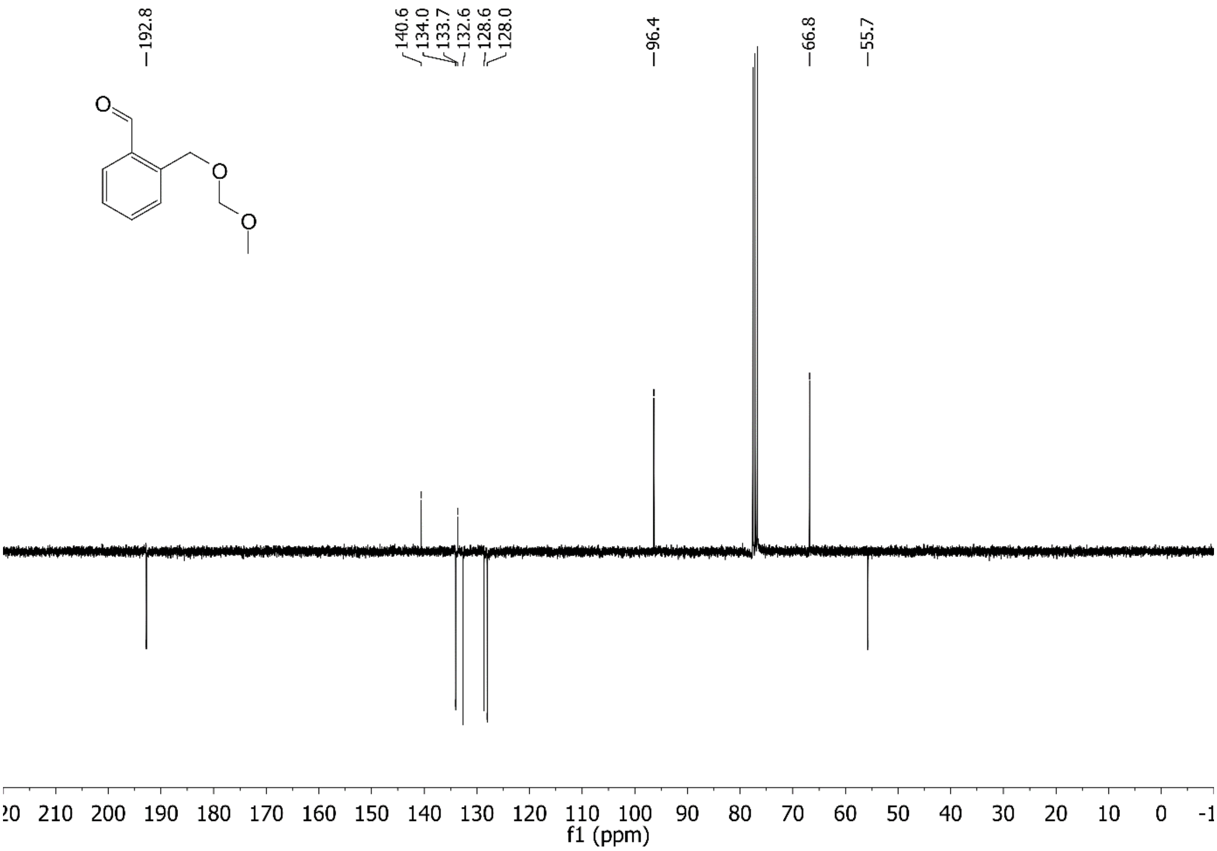

^{1}H NMR of **HM-SiR** (CD_3CN)

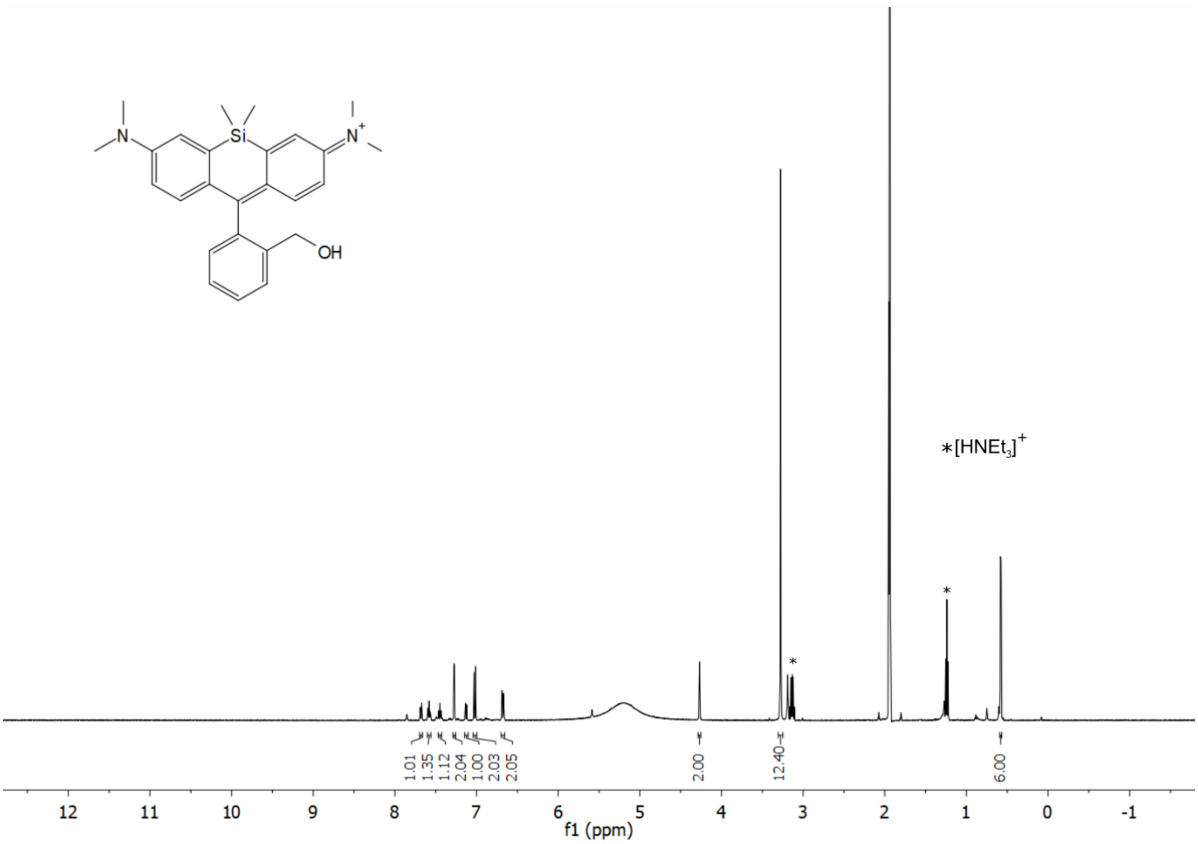

^{13}C NMR of **HM-SiR** (CD_3CN)

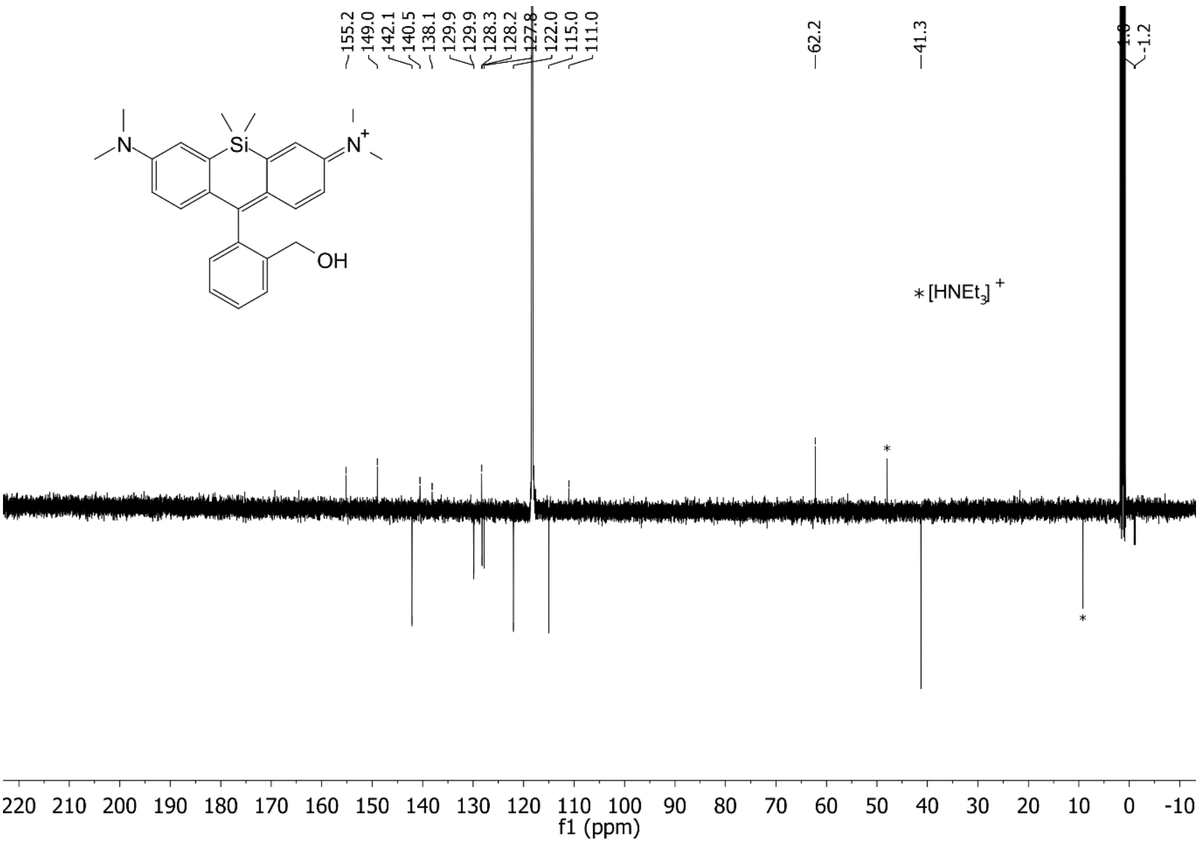

^{1}H NMR of **HTL-TCO*** (CD_3OD)

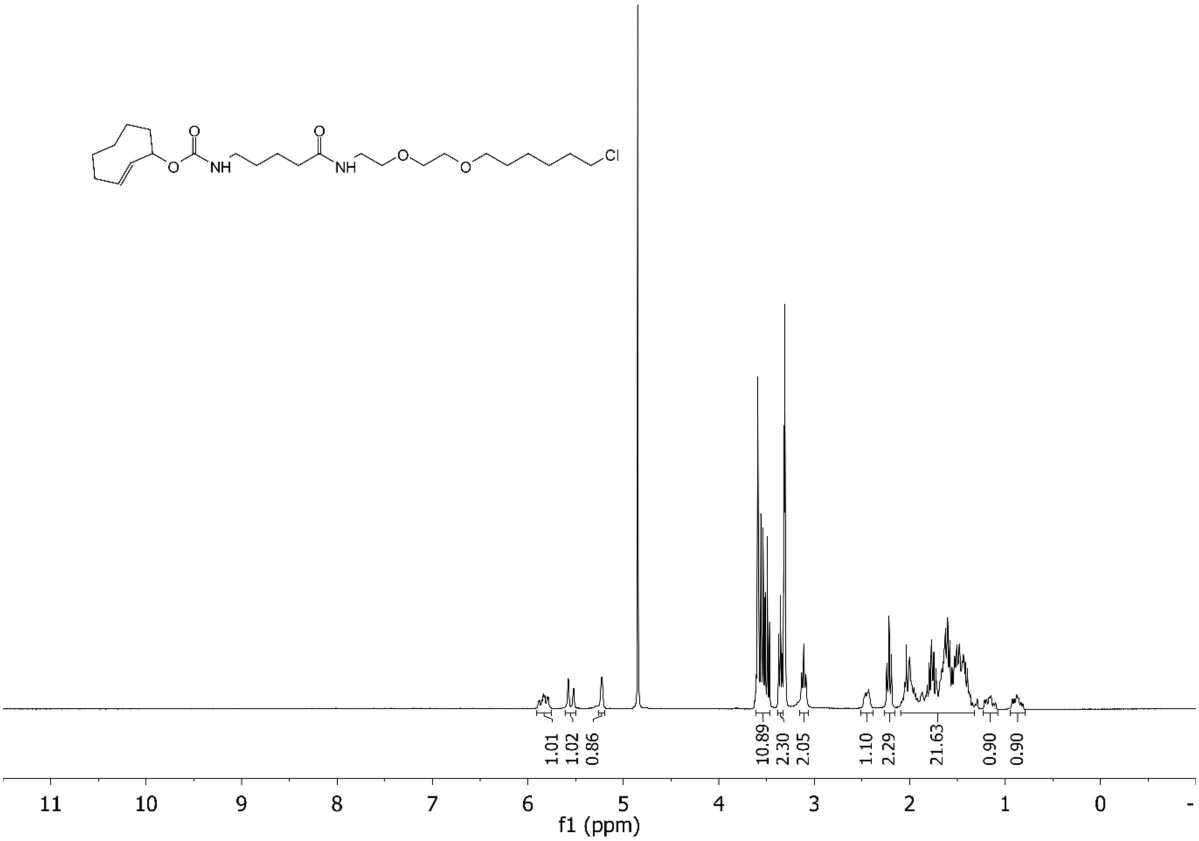

^{13}C NMR of **HTL-TCO*** (CD_3OD)

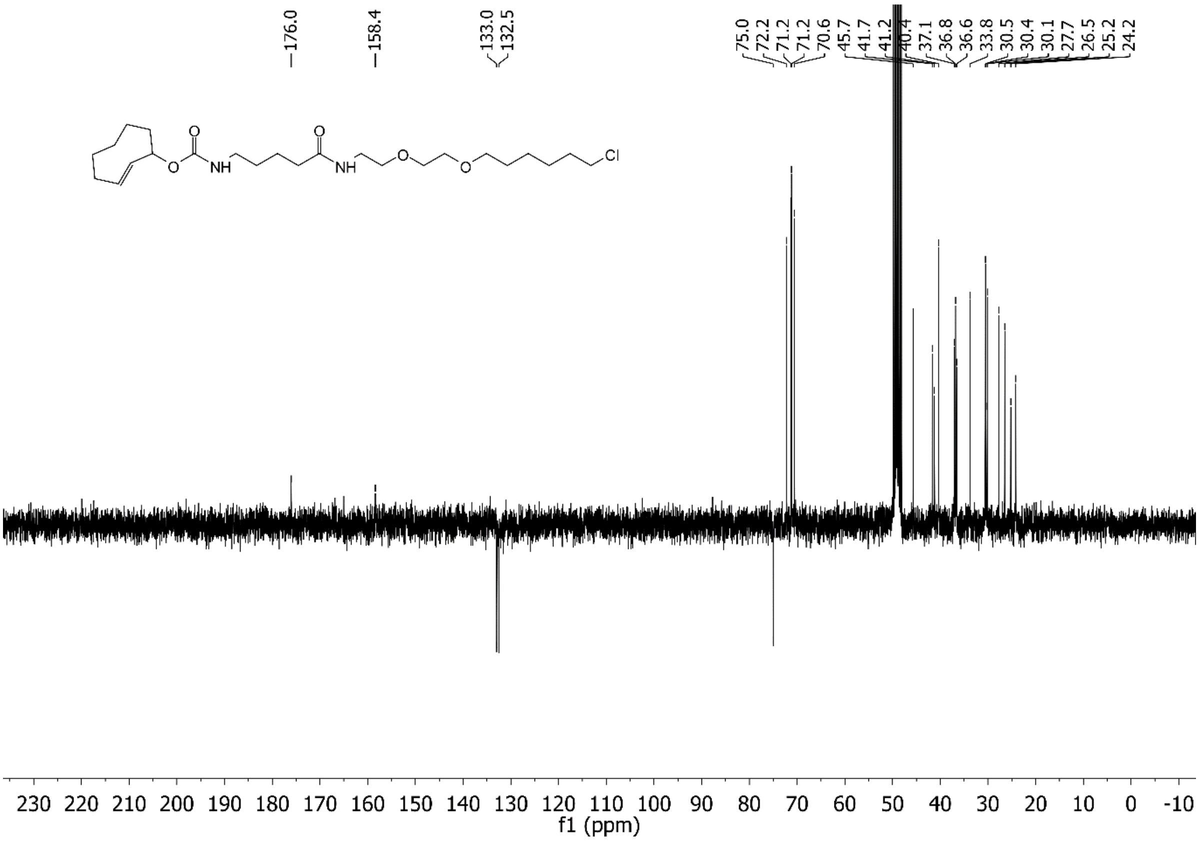

^{1}H NMR of **TPP-BCN** (CD_3OD)

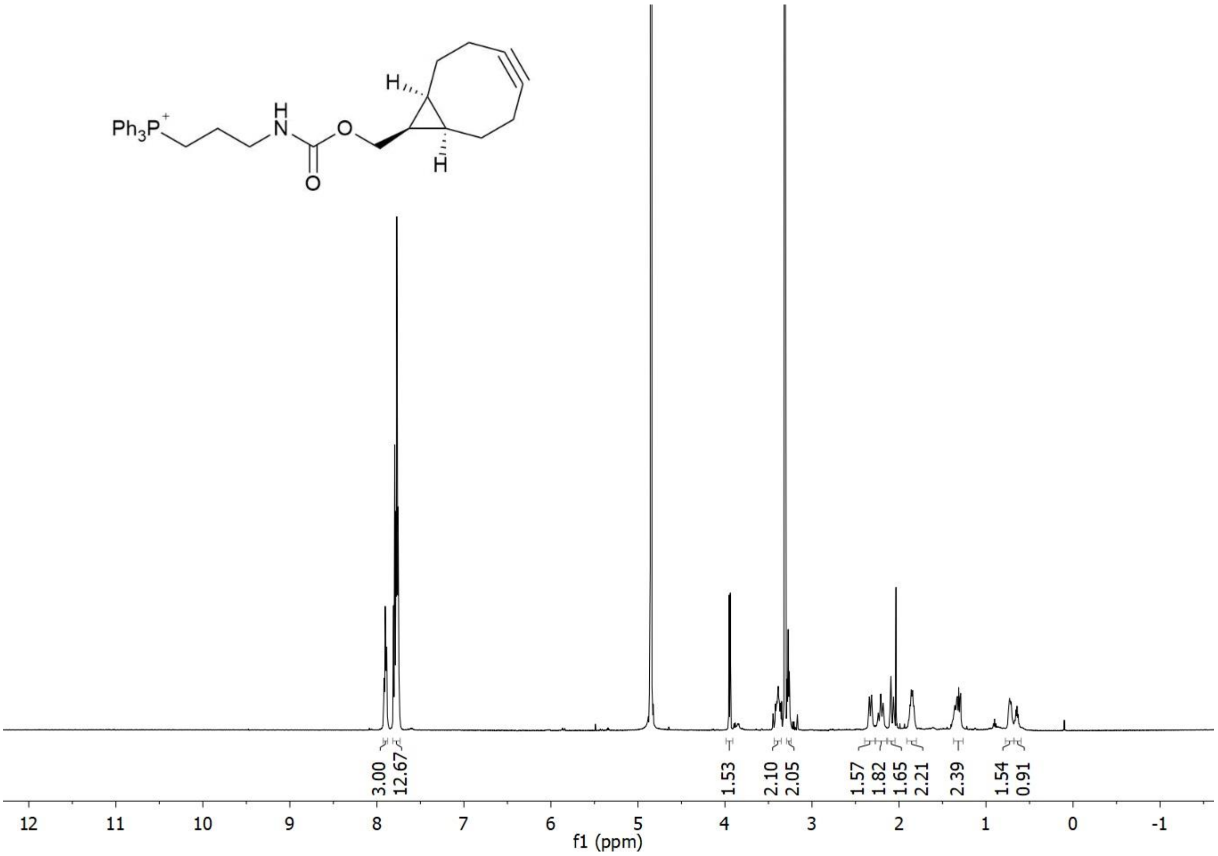

16 References

[1] G. R. Fulmer, A. J. M. Miller, N. H. Sherden, H. E. Gottlieb, A. Nudelman, B. M. Stoltz, J. E. Bercaw, K. I. Goldberg, *Organometallics* **2010**, *29*, 2176-2179.

[2] A. Brouwer, *Pure Appl. Chem.* **2011**, *83*, 2213-2228.

[3] C. Kitamura, N. Taka, T. Kawase, *Research on Chemical Intermediates* **2013**, *39*, 139-146.

[4] C. Aissa, A. Furstner, *J. Am. Chem. Soc.* **2007**, *129*, 14836-14837.

[5] J. Zanon, A. Klapars, S. L. Buchwald, *J. Am. Chem. Soc.* **2003**, *125*, 2890-2891.

[6] a) R. Selvaraj, J. M. Fox, *Tetrahedron Lett.* **2014**, *55*, 4795-4797; b) J. Yang, M. R. Karver, W. Li, S. Sahu, N. K. Devaraj, *Angew. Chem., Int. Ed.* **2012**, *124*, 5312-5315.

[7] A. Wieczorek, P. Werther, J. Euchner, R. Wombacher, *Chem. Sci.* **2017**, *8*, 1506-1510.

[8] H. E. Murrey, J. C. Judkins, C. W. am Ende, T. E. Ballard, Y. Fang, K. Riccardi, L. Di, E. R. Guilmette, J. W. Schwartz, J. M. Fox, D. S. Johnson, *J. Am. Chem. Soc.* **2015**, *137*, 11461-11475.

[9] M. Millard, J. D. Gallagher, B. Z. Olenyuk, N. Neamati, *J. Med. Chem.* **2013**, *56*, 9170-9179.

[10] G. Candiano, M. Bruschi, L. Musante, L. Santucci, G. M. Ghiggeri, B. Carnemolla, P. Orecchia, L. Zardi, P. G. Righetti, *Electrophoresis* **2004**, *25*, 1327-1333.

[11] M. Tokunaga, N. Imamoto, K. Sakata-Sogawa, *Nat. Methods* **2008**, *5*, 159.

[12] A. D. Edelstein, M. A. Tsuchida, N. Amodaj, H. Pinkard, R. D. Vale, N. Stuurman, *Journal of Biological Methods; Vol 1, No 2 (2014)* **2014**.

[13] S.-n. Uno, M. Kamiya, T. Yoshihara, K. Sugawara, K. Okabe, M. C. Tarhan, H. Fujita, T. Funatsu, Y. Okada, S. Tobita, Y. Urano, *Nat. Chem.* **2014**, *6*, 681.

[14] R. J. Marsh, K. Pfisterer, P. Bennett, L. M. Hirvonen, M. Gautel, G. E. Jones, S. Cox, *Nat. Methods* **2018**, *15*, 689-692.

[15] M. Ovesný, P. Křížek, J. Borkovec, Z. Švindrych, G. M. Hagen, *Bioinformatics* **2014**, *30*, 2389-2390.

[16] J. Schindelin, I. Arganda-Carreras, E. Frise, V. Kaynig, M. Longair, T. Pietzsch, S. Preibisch, C. Rueden, S. Saalfeld, B. Schmid, J.-Y. Tinevez, D. J. White, V. Hartenstein, K. Eliceiri, P. Tomancak, A. Cardona, *Nat. Methods* **2012**, *9*, 676.
